# Measurement of Adverse Events in Studies of Digital Health Interventions for Psychosis: Guidance and Recommendations Based on a Literature Search and Framework Analysis of Standard Operating Procedures

**DOI:** 10.1093/schbul/sbae048

**Published:** 2024-04-29

**Authors:** Emily Eisner, Cara Richardson, Neil Thomas, Mar Rus-Calafell, Suzy Syrett, Joseph Firth, Andrew Gumley, Amy Hardy, Stephanie Allan, Thomas Kabir, Thomas Ward, Aansha Priyam, Sandra Bucci, the iCharts network, Inez Myin-Germeys, Ulrich Reininghaus, Imran Chaudhry, Mario Alvarez, John Gleeson, Eric Granholm, Matthias Schwannauer, Philippa Garety, John Torous, Matteo Cella, Imogen Bell, Evelyne van Aubel, Tayyeba Kiran, Anita Schick, Xiaolong Zhang

**Affiliations:** Division of Psychology and Mental Health, School of Health Sciences, Faculty of Biology, Medicine and Health, Manchester Academic Health Sciences Centre, The University of Manchester, Manchester, UK; Research and Innovation, Greater Manchester Mental Health NHS Foundation Trust, Manchester, UK; Division of Psychology and Mental Health, School of Health Sciences, Faculty of Biology, Medicine and Health, Manchester Academic Health Sciences Centre, The University of Manchester, Manchester, UK; Centre for Mental Health and Brain Sciences, Swinburne University of Technology, Melbourne, Australia; Monash Alfred Psychiatry Research Centre, Alfred Hospital, Melbourne, Australia; Mental Health Research and Treatment Centre, Ruhr-Universität Bochum, Bochum, Germany; School of Health and Wellbeing, University of Glasgow, Glasgow, UK; NHS Research Scotland Mental Health Network, Edinburgh, UK; Division of Psychology and Mental Health, School of Health Sciences, Faculty of Biology, Medicine and Health, Manchester Academic Health Sciences Centre, The University of Manchester, Manchester, UK; Research and Innovation, Greater Manchester Mental Health NHS Foundation Trust, Manchester, UK; School of Health and Wellbeing, University of Glasgow, Glasgow, UK; Institute of Psychiatry, Psychology, and Neuroscience; King’s College London, London, UK; South London & Maudsley NHS Foundation Trust, London, UK; School of Health and Wellbeing, University of Glasgow, Glasgow, UK; McPin Foundation, London, UK; Departments of Experimental Psychology & Psychiatry, Oxford University, Oxford, UK; Institute of Psychiatry, Psychology, and Neuroscience; King’s College London, London, UK; South London & Maudsley NHS Foundation Trust, London, UK; Division of Psychology and Mental Health, School of Health Sciences, Faculty of Biology, Medicine and Health, Manchester Academic Health Sciences Centre, The University of Manchester, Manchester, UK; Research and Innovation, Greater Manchester Mental Health NHS Foundation Trust, Manchester, UK; Division of Psychology and Mental Health, School of Health Sciences, Faculty of Biology, Medicine and Health, Manchester Academic Health Sciences Centre, The University of Manchester, Manchester, UK; Research and Innovation, Greater Manchester Mental Health NHS Foundation Trust, Manchester, UK

**Keywords:** psychosis, schizophrenia, adverse effects, digital health, safety, harms

## Abstract

**Background:**

Given the rapid expansion of research into digital health interventions (DHIs) for severe mental illness (SMI; eg, schizophrenia and other psychosis diagnoses), there is an emergent need for clear safety measures. Currently, measurement and reporting of adverse events (AEs) are inconsistent across studies. Therefore, an international network, iCharts, was assembled to systematically identify and refine a set of standard operating procedures (SOPs) for AE reporting in DHI studies for SMI.

**Design:**

The iCharts network comprised experts on DHIs for SMI from seven countries (United Kingdom, Belgium, Germany, Pakistan, Australia, United States, and China) and various professional backgrounds. Following a literature search, SOPs of AEs were obtained from authors of relevant studies, and from grey literature.

**Results:**

A thorough framework analysis of SOPs (n = 32) identified commonalities for best practice for certain domains, along with significant gaps in others; particularly around the classification of AEs during trials, and the provision of training/supervision for research staff in measuring and reporting AEs. Several areas which could lead to the observed inconsistencies in AE reporting and handling were also identified.

**Conclusions:**

The iCharts network developed best-practice guidelines and a practical resource for AE monitoring in DHI studies for psychosis, based on a systematic process which identified common features and evidence gaps. This work contributes to international efforts to standardize AE measurement and reporting in this emerging field, ensuring that safety aspects of DHIs for SMI are well-studied across the translational pathway, with monitoring systems set-up from the outset to support safe implementation in healthcare systems.

## Introduction

The past decade has seen rapid development, worldwide, of digital health interventions (DHIs) for people with severe mental health problems, including schizophrenia-spectrum psychosis.^1–5^ Despite accumulating evidence of feasibility and acceptability of DHIs, information about their safety is not always systematically collected.^6,7^ When evaluating DHIs, it is vital to measure and collect empirical data on potential harms as well as potential benefits.^8,9^ Patients, healthcare professionals, policymakers, regulators, and funding bodies need this information when deciding whether to use, recommend, certify, prescribe, and/or fund a specific intervention. There is currently no internationally recognized guidance on how to measure, monitor, and record adverse events (AEs) associated with DHIs specifically.

Existing guidelines (summary: supplementary table 1) focus largely on how to monitor and report harms in pharmacological trials^7,10^; reporting of harms remains generally poor in DHI trials,^7^ psychological interventions,^10^ and behavioral/lifestyle interventions,^10^ all of which are relevant to schizophrenia-spectrum psychosis. As the field of digital health for psychosis moves beyond feasibility studies into larger trials testing DHI efficacy and effectiveness,^11^ and ultimately implementation (eg, SlowMo2), it is timely to harmonize measurement and reporting practices, ensuring future studies systematically monitor and record important DHI safety information. The current article addresses the need for resources and detailed best-practice guidance on AE measurement, monitoring, recording, and safety reporting in the context of DHIs for psychosis. Specifically, we examine how the international guidelines have been operationalized, to date, in this context, to describe and build on current methods, identify best practice, and make recommendations for future research.

Recent reviews^7,10^ examined AE reporting in published nonpharmacological studies, including those evaluating DHIs for psychosis, which may present particular challenges. However, reviewed articles provided scant information regarding studies’ overall AE procedures or how practicalities of measuring, eliciting, and recording AEs were managed.^7,10^ Both reviews highlighted several inherent difficulties in applying existing guidelines from the pharmacological literature to nonpharmacological trials, particularly regarding assessment of seriousness, relatedness, and expectedness. Given these challenges, and the limited reporting in peer-reviewed publications, it is difficult to make best-practice recommendations without access to more detailed information. Therefore, we undertook an in-depth, critical analysis of AE monitoring in current research practice, examining AE standard operating procedures (SOPs) obtained directly from study authors and from grey literature searches.

### Study Aims and Objectives

Aim: to develop and disseminate best-practice recommendations and a practical resource to support AE measurement, monitoring, recording, and safety reporting in DHI studies for people with psychosis.

Objectives: (1) to perform a framework analysis of detailed AE SOPs from existing digital psychosis studies and relevant national organizations; (2) to identify best practice in this context and to make recommendations enabling future digital psychosis studies to measure, monitor and report AEs consistently; and (3) to produce a template AE SOP which researchers can adapt to use in future digital psychosis studies.

## Methods

### Setting: The iCharts Network

The iCharts network (International Collaboration for Harmonising AE Reporting in Technology for Serious Mental Health Problems) is a group of international experts on DHIs for psychosis, with members from seven countries (United Kingdom, Belgium, Germany, Pakistan, Australia, United States, and China), including two low- or middle-income countries (LMIC; Pakistan and China). The network was convened by Prof Sandra Bucci and comprises a mixture of individuals with lived experience of psychosis (n = 3), senior researchers (n = 10), and early career researchers (n = 11). There were no specific selection criteria regarding countries that members could be from, but efforts were made to ensure at least one LMIC was represented. The network was supported by the Schizophrenia International Research Society’s (SIRS) 2021–2023 Research Harmonisation Award.

The iCharts network drew on existing datasets and members’ international expertise, taking particular care to consider cross-cultural issues, international differences, and lived experience perspectives. Three network members with lived experience of psychosis attended all iCharts network meetings, actively contributing to discussions about the overall direction of the program of work, and the network’s aims, methods, management, and outputs. We established two writing groups to address different aspects of our harmonization aim. Writing Group 1 collected raw AE data from existing digital psychosis studies to catalog the nature and range of AEs reported in this context. Writing Group 2 collated and analyzed AE SOPs (the focus of this article). An individual with lived experience of psychosis was a core Writing Group member, attending all meetings and contributing extensively to discussions about the work (including aims, methods, analysis, outputs, and dissemination) and to writing final study outputs.

### Phase 1: Search of Published Literature, Request to Study Authors, and Grey Literature Search

#### Search of Published Literature and Request to Study Authors

We searched seven databases (MEDLINE, PsycINFO, PsycARTICLES, Embase, Health and Psychosocial Instruments, PubMed, and Web of Science), combining search terms relating to psychosis/schizophrenia and digital health (supplementary method M1). As most digital health studies for psychosis were published since 2010,^12^ searches were restricted to peer-reviewed articles published in English since January 2010. Inclusion criteria were studies testing DHIs aiming to monitor/improve the mental/physical health of people with a psychosis or schizophrenia-spectrum diagnosis using a digital device (eg, smartphone app, text messaging, online/website, virtual reality, and wearable). We excluded studies where digital tools: (1) were used during in-person sessions only with no remote use (except VR studies, which were included based on in-person use only); (2) were used purely for research purposes; (3) only included video/phone calls; (4) were appointment booking systems; (5) were electronic health records (EHRs) that only health professionals could view/edit; (6) were only used to screen for mental health conditions; and (7) harvested existing data from EHRs or mainstream social media to predict/classify mental health conditions. Emily Eisner combined search results, removed duplicates, and screened titles and abstracts of retrieved results against eligibility criteria. Aansha Priyam independently screened the titles and abstracts of 10% of retrieved articles (randomly selected), with ratings then compared and disagreements resolved by consensus. Two researchers (Emily Eisner and Aansha Priyam) then independently screened the full text of the remaining articles against PICO criteria (supplementary method M2), with ratings compared and disagreements resolved by consensus.

Sandra Bucci contacted corresponding authors of all articles (n = 169) meeting inclusion criteria using a standard email template, citing the relevant article and requesting the SOPs/procedure/guide authors used to collect AE data in that study. Authors agreeing to provide this were directed to upload the information via a web-based proforma using the Qualtrics survey system (supplementary method M3). Authors who did not respond to the initial email request were prompted twice more at fortnightly intervals. SIRS also publicized the study to their members via email; however, no additional studies were identified via this route.

#### Grey Literature Search

In April 2022, we searched the grey literature (via Google search engine) for publicly available AE guidance. General searches, using multiple combinations of relevant search terms (eg, “harms,” “adverse events,” “medical device,” “safety reporting,” “clinical trials”), were supplemented by adding more targeted search terms relating to known funders of clinical research (eg, National Institute for Health and Care Research, Medical Research Council, Wellcome, National Health and Medical Research Council, National Institute of Health, National Institute of Mental Health), or relevant national regulators, agencies or governmental departments (eg, Health Research Authority, Medicines and Healthcare products Regulatory Agency, Food and Drug Administration, Office for Human Research Protections, Veterans Affairs, European Medicines Agency). Documents that were publicly available online and provided guidelines, recommendations, SOPs, or resources outlining how to elicit, measure, monitor, record, or report AE data during clinical trials were included. Unlike the peer-reviewed literature search, we did not limit the included documents to digital psychosis studies alone so as not to exclude national documents with a broader focus.

### Phase 2: Framework Analysis of the Standardized Operating Procedures

Documents collated during Phase 1 from DHI studies (n = 15), funders or regulators (n = 12), and UK NHS Trusts or universities (n = 5) were analyzed. To address the first two study objectives simultaneously (ie, analyze the existing SOPS; produce a template SOP), we selected the most comprehensive SOP retrieved (EMPOWER study^13^) and used this as the basis for an a priori coding framework for remaining SOPs and to provide the initial structure of the template SOP. The EMPOWER study SOP was the only included SOP where its intervention was registered in advance as a medical device. As such, it gathered more extensive safety information than other retrieved studies. By using the existing headings/subheadings of the EMPOWER SOP to create our a priori coding framework, and as the initial headings/subheadings of the template SOP, we were able to deductively code the contents of other SOPs into the framework and simultaneously draft an overall collated template SOP. Hence, the initial structure for the deductive coding came from the EMPOWER SOP, but content and examples were drawn from across all SOPS and synthesized during the analysis. Furthermore, while our coding was mainly deductive, we were also able to code inductively in instances where headings/subheadings from other SOPs were not present in the EMPOWER SOP. This combination of deductive and inductive coding is in line with a standard framework analysis approach.^14^ Further details of data analysis and synthesis are provided in supplementary method M4. Emily Eisner and Cara Richardson completed an initial round of coding of all SOPs, circulated to the wider group for feedback, and then adjusted the initial coding accordingly.

### Phase 3: Request for Additional Information From Study Authors

#### Additional Information on AE Procedures

From initial coding, it was evident that study SOPs did not fully document the details of all procedures of relevance to AE monitoring and recording. We anticipated that these procedures were in place, in practice, but simply not documented within the SOP itself. Therefore, where details of procedures were lacking, we re-approached study authors to request further information (supplementary method M5 for email template and question list). To maximize responses, we encouraged authors to submit additional information in whatever format was easiest for them. To increase the chances of sourcing relevant information, we also requested this information from the wider SIRS network.

#### Additional Information on AE Definitions

Although many study SOPs included definitions of AEs and their relevant subtypes, the subset of studies that did so was skewed towards UK studies. To increase the international relevance of the definitions included in our final SOP template, we requested AE definitions from iCharts members (or colleagues) in six further countries: China, Pakistan, India, Germany, Belgium, and the United States. We received AE definitions from all but one.

### Phase 4: Consolidation of Findings and Development of Final Template SOP and Recommendations

Having gathered additional information during Phase 3, authors Emily Eisner and Cara Richardson completed a final coding round, including these extra documents. Codes were then consolidated into a final draft template SOP for digital psychosis studies and circulated to the wider study team for feedback. In discussion with experts from across the iCharts network, we distilled key recommendations for AE reporting in future digital psychosis studies and devised a strategy for dissemination of the template SOP.

## Results

### Literature Searches and Requests to Study Authors

**Fig. 1.**
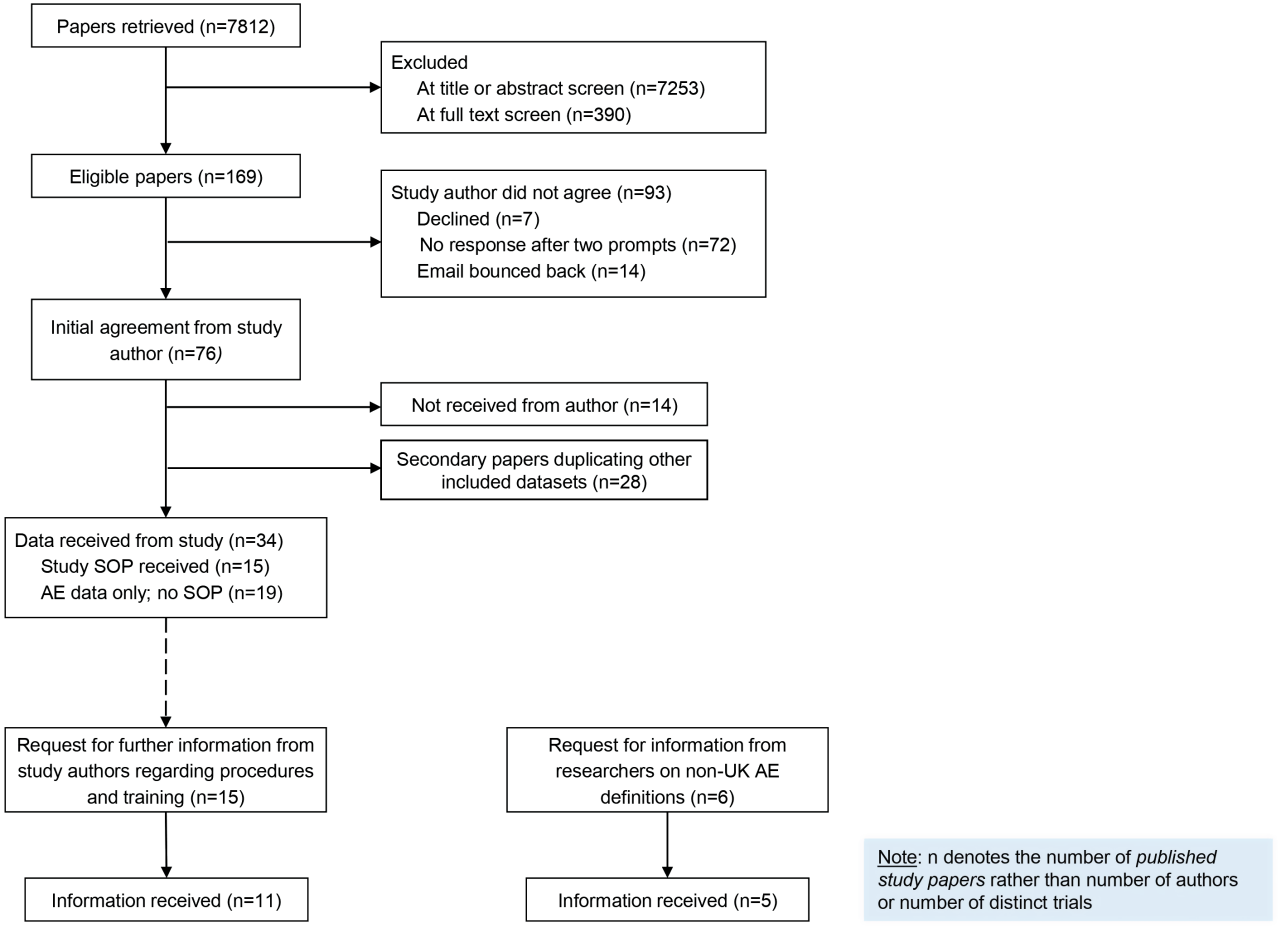
Flow diagram of literature search and requests for information from study authors.

Figure 1 outlines the number of retrieved and eligible articles, study authors approached, and study authors providing an AE SOP (n = 15; 10% of identified primary papers). Studies were from seven countries (Australia, Belgium, Canada, Netherlands, Spain, United Kingdom, and United States), tested a variety of DHIs (active symptom monitoring app, self-management app, cognitive behavior therapy (CBT)-informed app, passive sensing app, virtual reality, computerized cognitive remediation, computer-mediated therapy for distressing voices, online social therapy, digital reasoning intervention) and levels of blending, across various study designs (full-scale RCT, feasibility RCT, cohort study, pilot study). Figure 1 also shows the flow of requests for further information during Phase 3. Additionally, the search of grey literature identified 17 publicly available documents from funders or regulators (n = 12) and UK NHS Trusts or universities (n = 5).

### Overview of Results in Relation to Study Objectives

#### Objective 1: Framework Analysis

The EMPOWER study AE SOP formed the basis of the initial framework, supplemented by inductive coding of sections from other SOPS. Table 1 outlines the final framework, with details provided below.

**Table 1.** Summary of the Final Framework and Best-Practice Recommendations for AE SOPs in Digital Psychosis Studies

SOP Section^a^	SOP Subsections: Recommended Content^a^	Considerations or Examples	Relevant Resources^b^
*Introduction*	*Outline the purpose and scope of the SOP*	For example, to ensure all research staff are aware of AE definitions, and how to record, report and review these.	
	*Specify when AEs are reportable in this study*	AE monitoring usually starts when a participant signs the consent form and ends when the exit the study. Some studies include a “washout period” (eg, 30 days) in which AEs are monitored for a set time after study exit.	
	*List guidelines or regulations that the study must comply with in terms of AE monitoring and reporting*	Consult the sponsor regarding which guidelines and regulations are relevant.	Supplementary table 2: guidelines and regulations, by country
	*List AE subtypes to be assessed in the study*	Consult specific guidelines and regulations and consider type of intervention (medical device, therapy, drug, etc.).	
	*State: assessment of causality and expectedness are key*		
Responsibilities specific to the SOP	List AE-related responsibilities of each individual or committee involved in the study	This may include: Chief Investigator (CI), Principal Investigator (PI), trial/project manager, study research staff, Trial/Project Management Group (TMG/PMG), Trial/Project Steering Committee (TSC/PSC), study sponsor, research site Research and Development (R&D) departments, Data Management & Ethics Committee (DMEC), and/or clinical trials unit (CTU).	
*Definition of terms*	*List definitions of all AE types that are relevant to this study.* Consider structuring this section in line with the following four questions, in line with Figure 2	Consult specific guidelines and regulations and consider type of intervention (medical device, therapy, drug, etc.) when deciding which AE types are relevant.	Figure 1: AE flow diagram Supplementary table 1: ICH/ICO definitions Supplementary table 3: Definitions across reviewed SOPs
	Is it an AE? • Relevant AE type(s): AE	Before the study starts • List any events that will not be counted as AEs in this study (eg, technical glitches, phone theft). • Consider operationalizing a threshold above which an untoward medical or psychological occurrence constitutes an AE (and below which it does not).	
	Is it serious? • Relevant AE type(s): SAE	In addition to standard SAE definitions consider: • Are there any events that do not fall into the categories listed but that should be considered as SAEs for the purposes of the trial? For example, crisis care, suicide attempt, self-harm, police involvement. • Are there any events that fall into the categories listed in standard SAE definitions (supplementary table 3) but that should not be considered as SAEs for the purposes of the trial? Any changes of this type should be agreed and documented before the study starts.	Supplementary table 1: ICH/ICO definitions Supplementary table 3: Definitions across reviewed SOPs
	Is it related? • Relevant AE type(s): (S)AR, (S)ADR, (S)ADE, (S)TRAE	Before the study starts, consider: • How will relatedness be assessed? • Will a scale be used to assess relatedness? • Which specific aspects of the study will relatedness be assessed for? For example, software, hardware, study assessments, medication, psychological therapy. • Who has independent oversight of relatedness assessment? For example, Data Monitoring and Ethics Committee (DMEC).	Supplementary table 4: examples of relatedness scales used in reviewed SOPs
	Is it expected? • Relevant AE type(s): UAR, SSAR, SUSAR, ASTRAE, ASADE, USADE	• List expected *(S)ARs/(S)ADRs/(S)ADEs/(S)TRAEs* before the study starts. • Consult Reference Safety Information, if available. • If no Reference Safety Information exists yet, consider evidence from previous studies using this specific DHI or similar DHIs (eg, pilot studies, proof-of-concept studies) when defining expected (S)ARs. • Note that expectedness is assessed in relation to the study intervention/procedures (not the condition). • If there are no expected *(S)ARs/(S)ADRs/(S)ADEs/(S)TRAEs*, state this in the SOP.	Allan S., Ward, T., Eisner, E., et al. Adverse events reporting in digital interventions for psychosis (in preparation)
Monitoring AEs	Eliciting AEs Specify how AEs will be elicited, when, and by whom	• Consider which combination of methods listed in table 2 will be suitable for eliciting AEs in the study. • Consider blinding: how will AEs be elicited without unblinding blinded research staff? • Consider blending: is the DHI standalone or blended with therapy? This will affect the choice of AE elicitation method. For example, a standalone DHI may need additional phone calls or online surveys to screen for AEs. • What specific questions will research staff ask to elicit AEs? Consult lived experience contributors on how these questions are worded.	Table 2: methods for eliciting AEs
	Training and supervision Explicitly describe AE training and supervision for study staff All trained staff to sign AE SOP log	• Who will need to be trained on AE procedures? • How will they be trained, and by whom? • How will they be supervised, and by whom? • How else will you keep AEs on the research team’s radar (eg, add as a standing item to team meeting agendas)?	Table 3: training and supervision recommendations
*AE safety reporting requirements*	*What are the local reporting requirements?* *Which AE types need reporting and to whom?*	• Depending on the study type, report SAEs and SUSARS (or equivalent) to one or more of the following: the study Sponsor, site research office, Data Monitoring and Ethics Committee (DMEC), Trial Steering Committee (TSC), regulatory authority (eg, MHRA, Therapeutic Goods Association), Institutional Review Board (IRB)/Research Ethics Committee (REC)/Human Research Ethics Committee (HREC), device manufacturer. • Consult the sponsor and ensure that study AE SOP is in line with sponsor’s AE reporting requirements. • Consult local/national guidelines and regulations. • If the study DHI is registered as a medical device, there may be additional reporting requirements.	Supplementary table 2: guidelines and regulations, by country
Reporting AEs in publications and reports	Outline how the AEs will be analyzed and reported in study outputs	• For RCTs: Report AEs in the main study outcome paper in line with CONSORT extension for reporting harms (10-item checklist). • Consider whether AEs will be presented using descriptive statistics only or whether any inferential statistics are appropriate	CONSORT harms checklist: http://www.consort-statement.org/extensions?ContentWidgetId=561
*Appendices*		Consider including as appendices to the AE SOP: • Examples of AEs: supplementary table 5. • Report forms: supplementary material 6 ◦ AE report form ◦ Device Deficiency report form ◦ AE log spreadsheet • Reporting flowchart: supplementary material 7. • Declaration by study staff: supplementary material 8. • Glossary of terms: supplementary material 9.	Supplementary materials 5–9

^a^Sections from the original EMPOWER SOP are shown in italics and additional sections from other SOPs are shown in plain text.

^b^In all cases, see also the full collated SOP, available at https://documents.manchester.ac.uk/display.aspx?DocID=72386.

#### Objective 2: Best-Practice Recommendations

Table 1 also outlines best-practice recommendations for AE monitoring in digital psychosis studies, based on our analysis of AE SOPS. Table 2 provides detailed recommendations regarding how to elicit AEs and Table 3 outlines training and supervision recommendations.

**Table 2.** Methods for Eliciting/Monitoring AEs

Method for Eliciting AEs	Summary of Best Practice
1. Record spontaneously reported AEs	Research staff receive training and supervision to recognize and record AEs mentioned by participants spontaneously (eg, during therapy sessions or in passing during conversations) and seek additional information needed to determine seriousness and relatedness.
2. Record AEs elicited during study questionnaires or interviews	Research staff are aware of specific study assessments (questionnaires or interviews) that may elicit AE reports. They know what AE information to record and what follow-up information to seek as needed. For example, the following types of assessments may elicit AEs: • Assessments of participants’ mental or physical health; • Assessments asking use of healthcare services; for example, health economics assessments, or hospital admissions assessed as an outcome; • Qualitative interviews asking about experiences of using a digital intervention; • Assessments of suicidal ideation and/or harm to self or others.
3. Screen casenotes for AE reports	Research staff screen participant casenotes for evidence of AEs and seek further information as needed from clinical staff or participants themselves. The research team would first need to seek formal consent from participants (eg, on study entry) to access casenotes for this purpose.
4. Ask the clinical team about AEs	Research staff gather information from relevant clinicians about whether AEs have occurred. This may be embedded in regular meetings already happening with the clinical team (eg, clinical liaison meetings or follow-up assessments with staff), or researcher may contact staff specifically to seek information about AEs.
5. Ask open question(s) about whether the participant has experienced any AEs	Researchers ask specific question(s) to elicit AEs at each study contact. Researchers may wish to involve PPI colleagues or lived experience researchers in deciding specific question(s) to ensure they are pertinent and acceptable. As examples, one or more of the following questions might be used: *• Have you had any concerns about [DHI name]? If so, what concerns?* *• Have you had any unwanted experiences while using [DHI name]?* *• Did using [DHI name] have any negative impact on your mental well-being and/or physical health? If so, what was the impact?* *• Did using [DHI name] cause you any other issues or problems? If so, what issues or problems did the app cause for you?* *• Have you had any concerns about taking part in the study? If so, what concerns?* *• Have you been admitted to hospital since we last spoke? What led up to this?* If AEs are reported, the researcher will ask follow-up questions as appropriate to assess seriousness and relatedness.
6. Use a structured checklist or questionnaire to monitor AEs	At each assessment point, researchers actively check for the occurrence of specific AEs using a questionnaire or structured checklist. Example structured checklist used by researchers in one UK study: • Physical, death, self-harm, serious violent incidents (victim), serious violent incidents (accused), referrals to crisis care, admission to psychiatric hospital during therapy, and other. Example questionnaire from a UK study (completed via online survey software): *• Did using [DHI name] have any negative impact on your mental well-being and/or physical health? If so, what was the impact?* *• Did using [DHI name] cause you any other issues or problems? If so, what issues or problems did the app cause for you?*

**Table 3.** AEs Training and Supervision Recommendations

Training and Supervision recommendation	Summary of Best Practice
1. Provide AE training for research staff	All research staff (research assistants, research therapists, etc.): • Attend GCP training; • Read the study-specific AE SOP; • Attend a training session covering the overarching principles of AE monitoring/reporting and key points from the study-specific AE SOP. This may include: ◦ AE definitions; ◦ How AEs will be elicited and recorded in this study; ◦ Examples of AEs to look out for in this specific study; ◦ Particular study assessments to be aware of that may elicit AEs; ◦ Follow-up information that may be needed to classify AE seriousness and relatedness; ◦ Any events that usually considered AEs but exempted in this particular study context; ◦ Responsibilities of individual staff members in relation to AEs; ◦ How to monitor AEs while maintaining the blind (in a blinded RCT); ◦ Formal AE reporting requirements and who carries this out. This formal training is then embedded and reinforced during interactions with study PI and other members of the research team.
2. Provide ongoing supervision for research staff in relation to AE procedures	• AE monitoring is a standing item on the agenda in research staff supervision sessions (individual and group supervision). • Research staff are asked to bring in all suspected AEs for discussion during supervision. Discussions may include how to categorize a particular AE, whether any additional information is needed, next steps for recording and reporting, etc. • Any relevant changes to the AE procedure or any learning points from the wider study team are passed on to research staff in supervision sessions.
3. Include AEs as a standing item on team meeting agendas	• AE monitoring and reporting is standing item on the agenda in team meetings. This can apply across various levels of the project’s organizational structure—for example, local site meetings, multisite trial coordinator meetings, clinical trial management committee meetings. • Depending on the study design and specific meeting type, this may need to be a blinded agenda item—that is, blinded research staff leave the meeting for the duration of the AE-related discussion. • Research staff bring suspected AEs for discussion with the rest of the team. The level of detail of this discussion will depend on the context of the meeting itself but may include how to categorize a specific AE (eg, borderline cases), whether any additional information is needed, next steps for recording and reporting, etc. • Any relevant updates or learning points are shared. For example, changes to the AE procedure, learning points from other study sites, updates from the wider study team, or comments from DMEC report.

#### Objective 3: Template AEs SOP

A full template SOP, collating our findings from across the framework analysis of SOPs, will be made available (https://documents.manchester.ac.uk/display.aspx?DocID=72386) under a Creative Commons Attribution (CC BY). This resource is intended to provide a starting point from which researchers can adapt to create an AE SOP for use in studies that include a DHI or other digital health tool.

### *Framework Analysis of SOPs* (Table 1)

#### Introduction to the SOP

The original EMPOWER SOP included an introductory section outlining the AE SOP’s purpose and scope (ie, to ensure all research staff is aware of AE definitions, and how to record, report and review these), specified when AEs are reportable in this study (ie, from when the consent form is signed), mentioned specific local, national, and/or international regulations and guidelines that the study must comply with (ie, Medical Devices Regulations 2002, ISO/FDIS 14155:2011 and Standards for Good Clinical Practice), listed the specific AE types relevant to the study and, finally, noted that assessment of causality and expectedness are of particular importance.

Only 4/15 other study SOPs (all UK studies) included an introduction, with all 5 UK NHS Trust/university SOPs including one. Like EMPOWER, all nine stated the SOP’s purpose and scope and two listed specific AE types relevant to the study. The AE types differed depending on whether the DHI was classified as a medical device (“adverse device effect,” “serious adverse device effect,” etc.) or not (“adverse reaction,” “serious adverse event,” etc.), and whether the study chose to classify relatedness by subtype (therapy related, assessment related, device related). All nine introductions listed relevant regulations or guidelines, the specifics of which varied across the SOPs; a full list of regulations/guidelines relevant to DHIs for psychosis (collated from SOPs and our expert network) is provided in supplementary table 2.

#### Responsibilities Specific to the SOP

This section was added to the framework during inductive coding. Of study SOPs, 4/15 included a specific responsibilities section and 4/5 UK NHS or university SOPS contained one. SOPs variously described the responsibilities of the study Chief Investigator (CI), Principal Investigator (PI), trial/project manager, other study staff, Trial/Project Management Group (TMG/PMG), Trial/Project Steering Committee (TSC/PSC), study sponsor, site R&D (Research and Development) department, Data Management and Ethics Committee (DMEC), and/or clinical trials unit (CTU). The SOPs that did not include a specific responsibilities section often described the responsibilities of these individuals or groups at other relevant points, distributed throughout the SOP. However, our expert group agreed that having a specific section summarizing each person or group’s responsibilities was helpful and would promote better practice.

#### Definition of Terms

##### Overview

Most study SOPs (11/15), all NHS/university SOPS (5/5), and 11/12 funder/regulator guidance documents provided at least one AE-related definition. Our analysis of these definitions was supplemented by five additional definitions supplied by researchers from additional countries (China, Belgium, Germany, India, and the United States) during Phase 3. Definitions provided in all 32 documents were systematically compared.

**Fig. 2.**
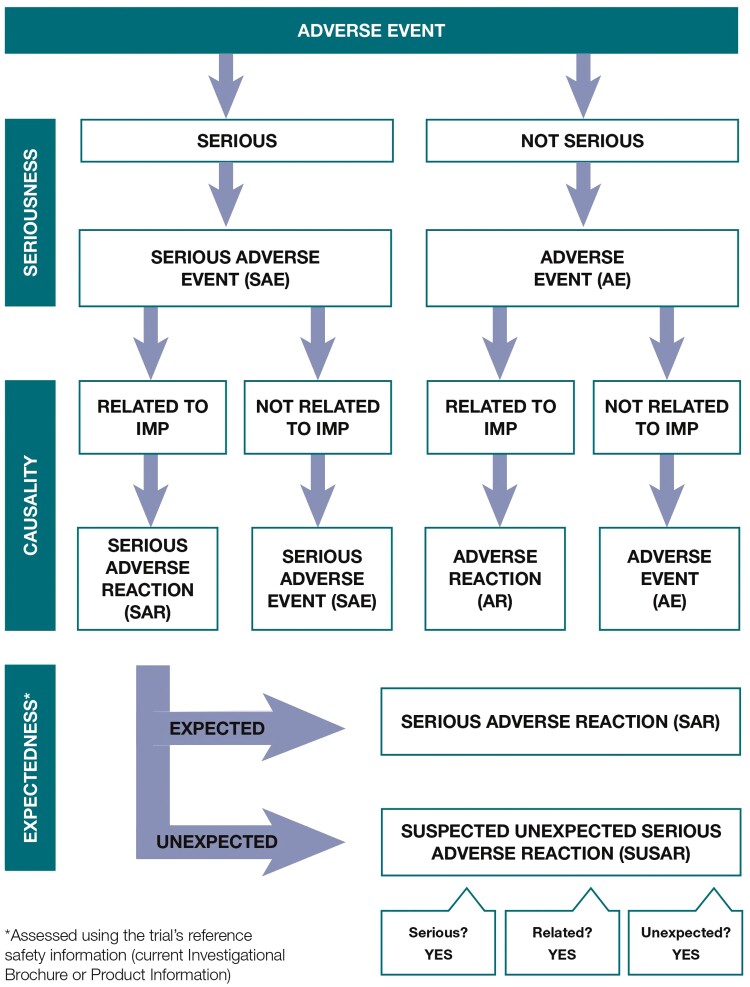
Safety reporting assessment flowchart for investigational medical products (IMPs), demonstrating a step-by-step approach to categorizing AEs. Reproduced with permission from the copyright holders (NIHR Clinical Trials Toolkit). An analogous process can be followed for categorizing AEs related to DHIs.

The SOPs/guidelines structured their description of AE definitions in various ways, with many providing a table or flow diagram in addition to a list of definitions. Experts in our network found the flow diagram from a publicly available guidance document (NHMRC guidelines, adapted from the NIHR Clinical Trials Toolkit) the most intuitive approach, so we structured our analysis of definitions in this way. This flow diagram is reproduced with permission from the copyright holder (NIHR Clinical Trials Toolkit) in Figure 2. Using the flow diagram, researchers answer the following questions to determine which definition applies to an event: Is it an AE? Is it serious? Is it related? Is it expected? This overall approach can be used across study types (eg, medical device, nonmedical device), albeit with changes to specifically named definitions. We compared each definition across SOPs, identifying core components and noting differences between SOPs, as shown in supplementary table 3 and outlined below.

##### Is it an AE?

Across sources, the core definition of AE was largely consistent and in line with ICH and ICO definitions (supplementary table 1), with three key components: a description of the type of event included (“any untoward medical or psychological occurrence…”), a statement of whom it applies to (“a study/trial participant…”), and a clarification that there does not necessarily need to be a causal relationship with study treatment or procedures. Supplementary table 3 outlines minor differences between definitions relating to these three components. We noted more substantial differences in two SOPs, from a Spanish study and a UK study. The former did not follow the usual structure but simply defined an AE as: “any event, action or behavior that is detrimental to the person’s recovery and/or hinders the delivery of the therapy.” Conversely, the UK study followed the usual structure but also operationalized a threshold above which untoward events would be defined as AEs: “[Events] which led to significant increased distress and interference with daily life such that intervention from clinical team is required… If this distress is managed by the trial team and does not require additional support from clinical services, then this is not classified as an adverse event.” Similarly, one other study noted specific events that would not be considered AEs (eg, “… technical glitches such as periodic network outage, other minor technical hitches with the apps, phone loss, phone theft and/or a participant selling the phone”) unless there was a resultant decline in mental state. Finally, 6/32 SOPS indicated that AEs would be classified by intensity (mild, moderate, and severe), with a slightly different subset (8/32 SOPs) clarifying that severity and seriousness are distinct concepts within AE procedures. With one exception (from the United States), all SOPs mentioning intensity/severity were from the United Kingdom.

##### Is it Serious?

The core definition of serious adverse event was largely consistent across sources (supplementary table 3), and in line with ICH and ICO definitions (supplementary table 1). All 24 definitions included clauses relating to death, life-threatening illness/injury, hospitalization, and persistent disability, and all but three (21/24) included a clause relating to effects on a fetus. Two final clauses, relating to medical/surgical intervention or other medically significant events, were less consistently included (13/24 and 9/24, respectively). There was no clear pattern of countries or types of study/guideline that included these. There were some variations in wording, and/or some definitions included specific additional notes on one or more of these clauses (supplementary table 3). Several definitions also noted that, depending upon the nature of the trial, the protocol may define certain events that do not fall into the categories listed but that should be considered as SAEs for the purposes of the trial. One UK study SOP specified that the following AEs would also be classified as serious: home treatment team involvement, suicide attempts, any violent incident necessitating police involvement (whether victim or accused), and self-harming behavior. Conversely, some definitions stated that the protocol may specify certain events that fall into the categories listed but that should not be considered as SAEs for the purposes of the trial.

##### Is it Related? (Causality)

Twenty-one SOPs provided a definition assessing the relatedness of an AE to one or more study elements, such as psychological therapy, medication, study procedure/assessment, and/or medical device. The following definitions were used in one or more SOPs (supplementary table 3):

- Adverse reaction (AR; 10 SOPs).
- Adverse drug reaction (ADR; 4 SOPs).
- Therapy- or assessment-related adverse event (TRAE; 1 SOP).
- Adverse device effect (ADE; 7 SOPs),

Each definition type had an equivalent to be used if seriousness criteria are met (supplementary table 3):

- Serious adverse reaction (SAR; 8 SOPs).
- Serious adverse drug reaction (SADR; 1 SOP).
- Serious therapy- or assessment-related adverse event (STRAE; 1 SOP).
- Serious adverse device effect (SADE; 7 SOPs).

To some extent, the definition type ((S)AR, (S)ADR, (S)TRAE, (S)ADE) depended on the specific element(s) being considered as potential causes of the (S)AE. If the (S)AE was related to a medical device, it was defined as an (S)ADE. If the (S)AE was related to any other study element, it could be defined as an (S)AR. However, one SOP defined (S)AEs related to the psychological therapy or study assessments as (S)TRAEs. Similarly, (S)AEs related to medication were either defined as (S)ARs or (S)ADRs. These differences were largely semantic, with the core definitions of (S)AR, (S)ADR, and (S)TRAE being similar (supplementary table 3).

Across definition types, some SOPs gave scant information about how to assess relatedness, simply stating that the (S)AE is “related to” or “a reaction to” the study element(s), whereas others used a more nuanced phrase like “reasonable possibility of a causal relationship” and 16 listed further detail in an additional section, entitled “causality.” Across SOPs, this section stated that the investigator should do one or more of the following when assessing relatedness: (1) use clinical judgment; (2) consider whether “there is evidence or argument to suggest a causal relationship”; (3) consider the timing of AE onset relative to the study element in question; (4) consider alternative causes such as the “natural history of the participant’s underlying condition, concomitant therapy, other risk factors, etc.”; (5) consult the current version of the risk analysis report and/or investigator’s brochure; (6) seek a second opinion from the DMEC/TMG regarding causality; and/or (7) use a scale to rate the AE’s likelihood of being related to the study element(s). Regarding the final point, 11 SOPs outlined details of a specific scale, typically a five-point scale (eg, definitely unrelated, probably unrelated, possibly related, probably related, definitely related); the definitions of each point on the scale differed somewhat (supplementary table 4).

##### Is it Expected (Anticipated)?

Twenty SOPs, mostly from the United Kingdom, provided at least one definition outlining the expectedness of the (S)AR (supplementary table 3):

- Unexpected adverse reaction (UAR; 7 SOPs).
- Suspected serious adverse reaction (SSAR; 1 study).
- Suspected unexpected serious adverse reaction (SUSAR; 8 SOPS).
- Anticipated serious therapy-related adverse event (ASTRAE; 1 study).
- Unanticipated serious therapy-related adverse event (USTRAE; 1 study).
- Anticipated serious device effect (ASADE; 3 studies).
- Unanticipated serious device effect (USADE; 3 studies).

Again, the exact type of definition used depended on the study element in question (psychological therapy, medication, study procedure/assessment, and/or medical device). Nevertheless, the core concept, across definition types, was that researchers should compare the (S)AR, or equivalent, against a list of expected (S)ARs to determine whether the AR was anticipated/expected or unanticipated/unexpected. For medication trials or medical device trials, SOPs explicitly referred to a list of expected (S)ARs in one of the following: reference safety information, investigator’s brochure, product information, summary of product characteristics, risk analysis report, clinical investigational plan, protocol, or risk assessment.

In study SOPs evaluating (S)ARs or equivalent relating to a DHI, psychological therapy, or study assessments, expectedness definitions varied more, and specific lists of expected ARs were rare. In fact, no study SOPs listed expected SARs, only two explicitly stated “no SARs are expected,” and only one study SOP listed specific ARs (“transient/short-lived increase in negative emotions [eg, some distress, tearful] during/on completing an assessment, interview or reviewing app content and consequent impact on functioning; minor irritation with app alerts/notifications”). Other expectedness definitions in study SOPs were vague, and many did not appear to require that ARs be prespecified; for example, one defined an unexpected AR as “an adverse reaction, the nature or severity of which is not consistent with the effects or consequences of what may be typically expected from a psychological intervention or completing the study assessments.” Conversely, one study SOP, which used the specific ASTRAE/USTRAE definitions, stated that expected ARs should be “identified prior to the commencement of the trial.” Similarly, a UK NHS Trust SOP emphasized the importance of listing expected events related to study interventions/procedures in advance, clarifying that “events which are not considered to be related to the study procedure do not need to be assessed for expectedness” and, further, that “expected does not mean: an event commonly seen in this patient population or in patients with this particular disease; a common side effect of surgery or other procedure; an event which causes no concern for the investigator.”

#### Monitoring AEs

##### Eliciting AEs

This section was added during inductive coding. Only four UK SOPs provided very basic information on procedure(s) used to elicit AE reports; namely, that researchers should actively ask participants at each study visit if they have experienced AEs. Only one SOP specified details: that a structured checklist would be used, and medical notes checked for extra AEs.

In Phase 3, study authors provided additional information indicating that a range of techniques for eliciting AEs were used in practice, albeit not formally documented in study SOPs. These techniques, summarized in table 2, included: recording spontaneously reported AEs, recording AEs elicited during non-AE-specific study assessments, screening casenotes for AE reports, asking the clinical team for AE information, asking participants open question(s) about whether they had experienced AEs, or using a structured checklist to elicit AEs from participants. Responses from study authors highlighted the importance of considering blinding during AE monitoring. In RCTs of blended DHIs, AE monitoring was often carried out by trial therapists rather than blinded research assistants. For standalone DHIs, a project manager or other unblinded staff member completed most AE monitoring.

##### Training and Supervision

Although most SOPs specified that certain AE monitoring tasks may be delegated to research staff (eg, research assistants, research therapists, and project managers), none specified how they would be trained or supervised. Again, we sought additional information from study authors on how they managed this in practice. Responses are summarized in table 3.

#### AEs Safety Reporting Requirements

25/32 SOPs outlined safety reporting requirements, the specifics of which varied depending on the country, sponsor organization type, research phase, and whether the DHI was registered as a medical device. Nevertheless, the principle behind these requirements was largely the same across SOPs; that is, that serious AEs must be reported to a particular institution, that reporting of certain events (eg, deaths) must be expedited, and that SUSARs require additional reporting. Institutions listed in relation to safety reporting were the study sponsor, site research office, Data Monitoring and Ethics Committee (DMEC), Trial Steering Committee (TSC), regulatory authority (eg, MHRA, Therapeutic Goods Association), Institutional Review Board (IRB)/Research Ethics Committee (REC)/Human Research Ethics Committee (HREC), and/or device manufacturer. Several SOPs included an additional note emphasizing that, when monitoring and reporting AEs, researchers should consider blinding but prioritize safety where unblinding is unavoidable.

#### Reporting AEs in Publications and Reports

Only one UK SOP specified how AEs would be analyzed and reported in the final study report: “For the final reports of the trial, the numbers, types, and severity of AEs by trial condition, as well as discontinuations, will be reported, using descriptive statistics. Since there are no prespecified hypotheses concerning AEs or harms, and, given the expected low frequency of AEs, the data will not be suitable for an Intention to Treat (ITT) statistical analysis.”

#### Appendices

Most SOPs included one or more appendices to aid implementation of AE procedures. Examples are provided in supplementary materials:

- Examples of AEs (supplementary table 5).
- Report forms/logs for AEs, Device Deficiencies, SAEs (supplementary material 6).
- Reporting flowchart (supplementary material 7).
- Declaration by study staff (supplementary material 8).
- Glossary of terms (supplementary material 9).

## Discussion

Our international network conducted an in-depth framework analysis of AE SOPs to develop best-practice guidelines (tables 1–3) and a practical resource to harmonize AE monitoring in DHI studies for psychosis. Our analysis revealed substantial gaps in AE SOPs regarding how AEs should be elicited and research staff training/supervision. We found a reliance on implicit knowledge about these processes rather than explicit documentation in SOPs. This is consistent with findings from recent reviews^7,10^ that published protocols and outcome papers lack information on AE procedures. Similarly, national guidelines tend to focus on definitions of AE subtypes, which AE subtypes should be reported, to whom, and within what timescales, rather than practicalities of eliciting AEs. We aim to make this implicit knowledge accessible by collating and publishing methods for eliciting/monitoring AEs (table 2) and training/supervision recommendations (table 3). A range of methods for eliciting AEs were used. The method selected for any given study may be influenced by factors such as DHI type, level of blending, study design, and sample characteristics. For example, studies using standalone or minimally blended DHIs should account for this lack of direct contact (eg, by including additional phone calls or online surveys to screen for AEs), and researchers conducting single-blind RCTs should consider how to manage blinding in relation to AE monitoring and reporting (eg, by allocating AE monitoring to unblinded researchers only, or by using a self-report mechanism for reporting AEs). Unlike double-blind medication studies, DHI study participants typically know which trial arm they are in; hence, certain aspects of AE monitoring (especially assessing relatedness) can potentially unblind researchers.

Core AE definitions were largely consistent across SOPs and in line with existing pharmacological guidelines, albeit with “investigational medicinal product” replaced by more relevant terms (eg, “digital health tool,” “investigational psychological therapy”). However, in line with recent reviews on the topic,^7,10^ our analysis suggested certain inherent difficulties of applying existing guidelines and definitions from the pharmacological literature to nonpharmacological trials in relation to defining seriousness, relatedness, and expectedness of AEs.

First, regarding seriousness, the priority for monitoring in pharmacological trials is on emergent symptoms arising from drug administration, particularly those that may cause lasting physical harm or threaten life (SAEs). With DHIs, which do not act directly on the body, the possibility of direct physical harm is much less of a priority for monitoring, and domains such as distress associated with use, and data protection may be more relevant. Nonpharmacological studies may miss potentially important harms if the study’s AE monitoring is restricted to SAEs (eg, hospital admission and death) rather than a broader range of AEs.^10^ Indeed, in a severe mental illness (SMI) population, there may be a relatively high base rate of unrelated hospital admissions, so focusing attention on monitoring SAEs alone may divert attention from more relevant harms which, although less serious initially, maybe no less important to the patient, and may have cumulative effects. For example, digital symptom monitoring is known to increase symptom awareness,^15^ which may trigger fear of relapse,^16^ which in turn can accelerate the relapse process.^17,18^ Further, unlike other interventions, DHIs are often embedded in participants’ daily lives which may cause additional stress, may dangerously interrupt daily activities (eg, driving), and could lead to violations of privacy if others access users’ personal data via devices. ARs to a DHI may also occur as an expected, temporary part of the therapeutic process; transparency about these can support users to make informed decisions about uptake and adherence to DHIs and prepare themselves to manage any transitory reactions.

Second, determining whether an AE is related to the intervention can be particularly challenging for nonpharmacological interventions.^10^ Although many SOPs provided definitions of relevant AE subtypes (eg, AR and ADE), they rarely gave sufficient information on how to actually assess relatedness. This is unsurprising given the difficulty in understanding the cause(s) of complex situations/events, particularly changes in human behavior and subjective well-being, which often result from multiple interacting dynamic factors. The most practical way that this issue was addressed in reviewed SOPs was by using a 5-point scale to operationalize relatedness assessment (supplementary table 4). In DHI studies, researchers should also define, before the study starts, the specific study elements for which AE-relatedness will be assessed (eg, software, hardware, study assessments, medication, and psychological therapy) and what sources may contribute relatedness information. Participants using a DHI may be able to supply valuable information about whether the DHI contributed to the development/maintenance of an AE (eg, whether they feel the DHI increased their focus on symptoms, accelerating relapse).

Third, unlike pharmacological trials, in which expectedness is determined by reference to detailed documentation of known effects from the drug manufacturer, it is difficult to list, a priori, all expected negative effects of a nonpharmacological intervention, as they may be novel and unknown.^7,10^ As figure 2 outlines, assessment of expectedness is only necessary for (S)AEs that are classed related to study intervention(s)/procedures. If no reference safety information exists yet, researchers may consider evidence from previous studies using the specific DHI or similar DHIs (eg, pilot/proof-of-concept studies). As DHIs with different overall aims, designs, or intended users often share common elements (eg, digital symptom monitoring, VR practice of skills learned in therapy), useful learning about expected AEs may be gleaned by examining a wide variety of literature. This underlines the importance of monitoring (S)AEs systematically in DHI studies, regardless of study phase, to gather information to add to the list of known (S)ARs. It is also crucial that this information is made publicly available so other researchers may learn from it in designing their own AE procedures. To support this, a wider aim of our international network’s work was to establish and publish a fine-grained analysis of the relative frequencies of AEs within digital psychosis trials.

Consistent with recent reviews,^7,19–21^ AE SOPs rarely specified an analysis and reporting plan. We suggest that SOPs should include a section specifying how AEs will be analyzed to ensure that AE data collection and storage allows adequate reporting. For example, the harms extension^22^ of the Consolidated Standards of Reporting Trials statement (CONSORT) provides 10 recommendations on best-practice reporting of AEs. Although it is standard practice to report AEs using descriptive statistics alone, methodologists have recently begun developing more nuanced ways of analyzing AEs in clinical trials,^23^ accounting for the relative infrequency of AEs and the likelihood of bias toward AE reporting in the intervention arm of an RCT.

As empirical evidence supporting the efficacy of DHIs for psychosis accumulates, it will become increasingly important to develop AE monitoring procedures regarding wider implementation of DHIs in health services, outside a clinical research context. Such procedures will likely depend on local/national governance processes, including those of regulatory agencies (eg, MHRA yellow card reporting scheme, EudraVigilance). Nevertheless, stakeholders would benefit from international consistency regarding how AE monitoring is managed and reported in the context of implementation, and extended international guidelines would be welcomed. Ultimately, it is essential that data on potential harms of DHIs is collected and made public, whether in a research context or during implementation, so that stakeholders (eg, patients, health professionals, policymakers, regulators, and funding bodies) can access this information when deciding whether to use, recommend, certify, prescribe and/or fund a specific DHI.

### Strengths and Limitations

We conducted an in-depth examination of 32 AE SOPS/guidelines, systematically analyzing their content, supported by an international network, including individuals with lived experience of psychosis. By searching the peer-reviewed and grey literature, we included AE SOPs from a wide range of contexts, including different countries, DHI types, technology types, levels of blending, study designs, funders, and organization types. Although EMPOWER (a medical device study) contributed to our recommendations more than other individual SOPs, content, and recommendations were drawn from across included SOPs. The main distinction between medical device study SOPs and those from studies testing a DHI not registered as a medical device was the list of relevant AE definitions. Hence, the core recommendations apply equally well to both types of studies and are based on a thorough analysis of a wide range of SOPs. Nevertheless, there were limitations. First, while our aim was to collate recommendations across SOPs in a way that can be used across contexts, certain aspects are necessarily context dependent. In particular, safety reporting requirements rely on local regulations and will therefore differ across countries. Second, both the published and grey literature searches were of English language documents only, biasing the results towards SOPS/guidelines of English-speaking countries. Similarly, although extensive, the published literature search was not fully comprehensive and we were reliant on study authors taking the time to send their SOPs, potentially introducing selection bias by omitting work related to AE reporting in other contexts/countries. The grey literature search was not systematic or comprehensive, with documents appearing higher in standard Google searches more likely to be included. Indeed, UK SOPs were over-represented in both cases. We recognize that our findings are influenced by the relatively small number of countries represented by our network’s membership. We welcome feedback and additional content from experts, including experts by experience, around the world, with an increased focus on nations not currently represented to maximize and broaden relevance of this guidance.

## Conclusions

The iCharts network carefully analyzed AE SOPs to develop best-practice guidelines for AE monitoring in DHI studies for psychosis, highlighting useful common features and evidence gaps. This process allowed us to develop a practical resource for AE monitoring and reporting, including training guidance and procedures. AE measurement in this field is likely to evolve over time as DHI studies for psychosis become widespread, and as common AEs are better documented and known. Our intention is for the publicly available template SOP to be a live document, updated at intervals as needed. Our findings contribute towards international efforts to standardize the measurement and reporting of AEs in the emerging field of digital mental health to ensure that the safety aspects of DHIs for SMI are well-studied in research and well-understood by those ultimately implementing such interventions.

## Supplementary Material

Supplementary material is available at https://academic.oup.com/schizophreniabulletin/.

sbae048_suppl_Supplementary_Materials

sbae048_suppl_Supplementary_Methods
