# Supplementary material for: Measurement of Adverse Events in Studies of Digital Health Interventions for Psychosis: Guidance and Recommendations Based on a Literature Search and Framework Analysis of Standard Operating Procedures
Source: Schizophr Bull. 2024 Apr 29;50(6):1456–70. doi: 10.1093/schbul/sbae048 (PMC11548926; doi:10.1093/schbul/sbae048)
Supplement: sbae048_suppl_Supplementary_Materials [file sbae048_suppl_supplementary_materials.docx]

**Supplementary Tables and Materials**

**Table S1. Definitions of Adverse Events and Serious Adverse Events from the International Council for Harmonisation (ICH) and International Organization for Standardization (IOS)^[[1]](#footnote-1)^**

|  | **International Council for Harmonization definition** | **International Organization for Standardization definition** |
| --- | --- | --- |
| Adverse Event (AE) | Any untoward medical occurrence in a patient or clinical investigation subject administered a pharmaceutical product and which does not necessarily have a causal relationship with this treatment. An adverse event (AE) can therefore be any unfavourable and unintended sign (including an abnormal laboratory finding), symptom, or disease temporally associated with the use of a medicinal (investigational) product, whether or not related to the medicinal (investigational) product.” (ICH, 2016, pg. 2). | Untoward medical occurrence, unintended disease or injury, or untoward clinical signs (including abnormal laboratory findings) in subjects, users or other persons, whether or not related to the investigational medical device.  Notes:  1. This definition includes events related to the investigational medical device or the comparator.  2. This definition includes events related to the procedures involved.  3. For users or other persons, this definition is restricted to events related to the use of investigational medical devices or comparators.” (ISO, 2020, Section 3.2) |
| Serious Adverse Event (SAE) | Any untoward medical occurrence that at any dose:   - - results in death,   - is life-threatening,   - requires inpatient hospitalization or prolongation of existing hospitalization,   - results in persistent or significant disability/incapacity, or   - is a congenital anomaly/birth defect | adverse event (3.2) that led to any of the following  a) death,  b) serious deterioration in the health of the subject (3.50), users, or other persons as defined by one or more of the following:  1) a life-threatening illness or injury, or  2) a permanent impairment of a body structure or a body function including chronic diseases, or  3) in-patient or prolonged hospitalization, or  4) medical or surgical intervention to prevent life-threatening illness or injury, or permanent impairment to a body structure or a body function,  c) foetal distress, foetal death, a congenital abnormality, or birth defect including physical or mental impairment  Note 1 to entry: Planned hospitalization for a pre-existing condition, or a procedure required by the CIP (3.9), without serious deterioration in health, is not considered a serious adverse event. |

**Table S2. Relevant links to legal content, regulations, and guidelines specific for digital health and medical devices (by region)**

| Country/Region | Resources |
| --- | --- |
| Worldwide | |
| Worldwide | - ISO 14155 (Clinical investigation of medical devices for human subjects — Good clinical practice)   <http://www.iso.org/iso/catalogue_detail?csnumber=45557> |
|  | - Deploying digital health tools within large, complex health systems: key considerations for adoption and implementation <https://www.nature.com/articles/s41746-022-00557-1> |
|  | - Global strategy on digital health 2020-2025 <https://www.who.int/docs/default-source/documents/gs4dhdaa2a9f352b0445bafbc79ca799dce4d.pdf> |
| Europe | |
|  | - General Data Protection Regulation (GDPR): <https://gdpr.eu/> |
| European Union (EU) | - European Regulation on Medical Devices: <https://eur-lex.europa.eu/legal-content/EN/TXT/?uri=CELEX%3A32017R0745> |
|  | - European Commission Medical Devices Guidance Document (MEDDEV 2.12-1 rev 8) <http://www.cepartner4u.com/wpdm-package/meddev-2-12_1-vigilance-system/> |
| Germany | - Federal Institute For Drugs and Medical Devices: <https://www.bfarm.de/EN/Medical-devices/Applications-and-reports/SAE-report/Clinical-investigation/_artikel.html> |
| England & UK | |
| England | - Evidence standards framework for digital health technologies: user guide <https://www.nice.org.uk/about/what-we-do/our-programmes/evidence-standards-framework-for-digital-health-technologies> |
|  | - UK GDPR Guidance <https://ico.org.uk/for-organisations/uk-gdpr-guidance-and-resources/> |
|  | - Digital Clinical Safety Strategy <https://transform.england.nhs.uk/key-tools-and-info/digital-clinical-safety-strategy/> |
|  | - Digital Technology Assessment Criteria: <https://transform.england.nhs.uk/key-tools-and-info/digital-technology-assessment-criteria-dtac/> |
| United Kingdom (UK) | - Health Research Authority - Safety Information   <http://www.hra.nhs.uk/research-community/during-your-research-project/safety-reporting/> |
|  | - A guide to good practice for digital and data-driven health technologies <https://www.gov.uk/government/publications/code-of-conduct-for-data-driven-health-and-care-technology/initial-code-of-conduct-for-data-driven-health-and-care-technology> |
|  | - MHRA - Medical devices: guidance for manufacturers on vigilance: <https://www.gov.uk/government/collections/medical-devices-guidance-for-manufacturers-on-vigilance> |
| Asia | |
| India | - Indian Pharmacopeia Commission <https://www.ipc.gov.in/> |
|  | - Pharmacovigilance Programme of India (PvPI) <https://www.ipc.gov.in//PvPI/pv_home.html> |
| America | |
| United States | - Food and Drug Administration (Medical Devices content): <https://www.fda.gov/medical-devices> |

**Table S3. Core definitions collated across studies/guidelines, and notes on variations**

| **Definition type** | **Core definition** | **Notes on variations or additions to core definition components** | **Definitions** |
| --- | --- | --- | --- |
| Is it an adverse event? | | | |
| Adverse event (AE) | 1. Any untoward medical or psychological occurrence, unintended disease or injury, or untoward clinical signs… 2. …in a study/trial participant (including those in an untreated control group). 3. Adverse events do not necessarily have a causal relationship with the study treatment or study procedures (but may do). | 1. Often slight wording variations. E.g., phrases such as “unfavourable and unintended sign”, “undesirable experience”, “harmful event”, or “abnormal lab findings”. 2. Control group/condition is not always explicitly included within the scope of the definition. Especially for definitions primarily used within double blind medication trial studies (e.g. EU clinical trials regulation; NHMRC guidelines), in which researchers logging AEs would not know group allocation. 3. Depending on the specifics of the study treatment, additional items are included in the final clause; e.g., “digital health tool”, “investigational psychological therapy”, “investigational medical device”, or “medicinal product”. Study procedures (as opposed to treatment) were not always explicitly included in the list. | Total: 26  UK: 14  Europe: 5  US: 3  China: 1  India: 1  Australia: 2 |
| Is it serious? | | | |
| Serious Adverse Event (SAE) | An Adverse Event is defined as serious if it:   1. Results in death or, 2. Is a life-threatening illness or injury or, 3. Requires [voluntary or involuntary] hospitalisation or prolongation of existing hospitalisation or, 4. Results in persistent or significant disability or incapacity or, 5. Consists of a congenital anomaly or birth defect [or leads to foetal distress or foetal death] or, 6. Necessitates medical or surgical intervention to prevent any of the above or, 7. Is otherwise considered medically significant by the investigator. | 1. Deaths occurring within 30 days of the last administration of the study treatment are SAEs 2. Participant was at risk of death at the time of the event (events that hypothetically might have caused death if it were more severe are not included). 3. Planned hospitalisation for a pre-existing condition, or a procedure required in the protocol, without a serious deterioration in health are not SAEs. 4. Alternative wording: “Results in a permanent impairment of a body structure or a body function”. 5. Some definitions do not state foetal distress/death. 6. -- 7. Medical and scientific judgement should be exercised. One   One study specified that the following would be considered SAEs: home treatment team involvement, suicide attempts, any violent incident necessitating police involvement (whether victim or accused), and self-harming behaviour. | Total: 24  UK: 14  Europe: 4  US: 3  China: 1  India: 1  Australia: 1 |

| Is it related? | | | |
| --- | --- | --- | --- |
| Adverse Reaction (AR) | An adverse event…   1. …judged by either the reporting investigator or the sponsor… 2. …as having a reasonable possibility of a causal relationship (e.g. definitely, probably or possibly related)… 3. …to the [psychological therapy, study procedure, therapy, other intervention, investigational medicinal product, drug, experiment]… 4. … related to any dose or duration of therapy administered to that subject. | 1. 4/10 mentioned who judges relatedness in the study, often stating “where there are two assessments of an event, the causality assessment made by the local investigator cannot be downgraded. In the case of a difference of opinion on causality, both assessments are recorded, and the “worst case” assessment is used for reporting purposes.” 2. 4/10 simply stated the AE “is related” or a “reaction to” study element(s); 6/10 used a more nuanced phrase like “reasonable causal relationship” or similar. 3 studies used a scale to define the likelihood of the AE being related to the study element. 3. All 10 SOPs specified that the AE is related to one or more of the stated elements. However, some of these elements (investigational medicinal product, study procedures) overlap with elements specified in other definitions (Adverse Drug Reaction and TRAE, respectively). 4. 5/10 definitions mentioned dose, 4 in the context of a medicinal product and 1 in relation to dose of a psychological therapy. | Total: 10  UK: 6  Europe: 2  US: 1  China: 0  India: 0  Australia: 1 |
| Serious Adverse Reaction (SAR) if meets seriousness criteria | | |  |
| Adverse Drug Reaction (ADR) | 1. An untoward reaction 2. … judged as having a reasonable probability of a causal relationship with…. 3. … a drug… 4. … at any dose | 1. Wording varied across definitions: untoward / unintended / harmful / noxious / unexpected 2. 2 definitions used the phrase “reasonably probability” or “reasonable possibility” and clarified that this meant there was “evidence or argument to suggest a causal relationship” or “an association cannot be ruled out”; 2 did not mention probability. 3. Sometimes described as “Investigational Medicinal Product” or “investigational drug” 4. Not all definitions mentioned dose. One definition specified different dose criteria for approved pharmaceutical products (“at doses usually used in humans”) and new unregistered pharmaceutical products (“any dose”) | Total: 4  UK: 1  Europe: 0  US: 0  China: 1  India: 1  Australia: 1 |
| Serious Adverse Drug Reaction (SADR) if meets seriousness criteria | | | |
| Therapy or Assessment Related Adverse Event (TRAE) | Adverse event as described in [AE definition] related to [study] therapy or [study] research assessment. | Only defined in one UK study. | Total: 1  UK: 1 |
| Serious Therapy or Assessment Related Adverse Event (STRAE) if meets seriousness criteria | | | |
| Adverse Device Effect (ADE) | AE related to the use of an Investigational Medical Device. This includes AEs resulting from insufficient or inadequate instructions for use, deployment, [implantation], installation, or operation, or any malfunction of the Investigational Medical Device. This definition includes any event resulting from use error or from intentional misuse of the Investigational Medical Device. Use error refers to an act or omission of an act that results in a different device response than intended by the manufacturer or expected by the user. | 6/7 definitions were virtually identical.  The remaining definition, from the glossary of a UK guideline, was tautological: “Adverse Device Event means a device-related adverse event” | Total: 7  UK: 6  Europe: 0  US: 0  China: 0  India: 0  Australia: 1 |
| Serious Adverse Device Effect (SADE) if meets seriousness criteria | | Two SOPs added: “This includes Device Deficiencies that might have led to a serious adverse event if: Suitable action had not been taken or, intervention had not been made or, if circumstances had been less fortunate” |  |
| Device Deficiency | Inadequacy of the medical device with respect to its identity, quality, reliability, safety or performance. Device Deficiencies include malfunctions, misuse, end user errors, inadequate labelling and inadequacy in the information supplied by the manufacturer. | One SOP specifies: “Some deficiencies may have led to an adverse device effect or a serious adverse device effect, and should be treated as ADEs/SADEs”. | Total: 4  UK: 4 |
| Is it expected? | | | |
| Unexpected Adverse Reaction (UAR) | An adverse reaction, the nature or severity of which is not consistent with…  … information known about the effects or consequences that may be typically expected from the psychological therapy/intervention being investigated or from completing the study assessments  OR  … available information on the investigational medicinal product in the Reference Safety Information, investigator’s brochure, Product Information, Summary of Product Characteristics, or equivalent (as applicable). | One SOP added: “in the view of the investigator” | Total: 7  UK: 4  US: 1  Australia: 1  Europe: 1 |
| Suspected Serious Adverse Reaction (SSAR) | An adverse reaction that is classed in nature as serious and which is consistent with the information about the medicinal product in question set out in the Summary of Product Characteristics or Investigator’s Brochure, as applicable |  | Total: 1  UK: 1 |
| Suspected Unexpected Serious Adverse Reaction (SUSAR) | An adverse event that is judged to be…  …Serious [see SAE definition]  …Related [see AR definition]  …Unexpected [see UAR definition] | The core SUSAR definition was the same across SOPs, with slight wording differences, mainly regarding whether the SUSAR definition fully listed the details of the three components (seriousness, relatedness, expectedness) or cross-referenced other definitions containing these components. | Total:  UK: 8  Europe: 2  Australia: 1  China: 1 |
| Anticipated Serious Therapy Related Adverse Event (ASTRAE) | A serious therapy or assessment related adverse event which by its nature, incidence, severity or outcome has been identified prior to the commencement of the trial.  EITHER: We do not anticipate any ASTRAEs  OR: The following ASTRAEs are anticipated: [list] |  | Total: 1  UK: 1 |
| Unanticipated Serious Therapy Related Adverse Event (USTRAE) | A serious therapy or assessment related adverse event which by its nature, incidence, severity or outcome has not been identified in [ASTRAE definition] |  | Total: 1  UK: 1 |
| Anticipated Serious Device Effect (ASADE) | 1. A Serious Adverse Device Effect (SADE) which by its nature, incidence, severity or outcome… 2. …has been previously identified in the [risk analysis report, Investigator’s Brochure, Clinical Investigational Plan, or risk assessment]… 3. EITHER: We do not anticipate any SADEs   OR: The following SADEs are anticipated: [list] | 1. – 2. Documents referred to here varied by SOP 3. Two study SOPs specified in the definition whether any SADEs are anticipated. | Total: 3  UK:3 |
| Unanticipated Serious Device Effect (USADE) | 1. A serious adverse device effect which by its nature, incidence, severity or outcome… 2. …has not been previously identified in the [risk analysis report, Investigator’s Brochure, Clinical Investigational Plan, protocol, or risk assessment]… | 1. – 2. Documents referred to here varied by SOP | Total: 6  UK: 5  Australia: 1 |

**Table S4. Scales used to assess the likelihood that an (S)AE is related to an element of the study**

|  | **2 UK NHS Trust SOPs** | **UK University SOP** | **UK NHS Trust SOP** | **2 UK study SOPs** | **US guideline** | **UK study SOP** |
| --- | --- | --- | --- | --- | --- | --- |
| **Not related**  **Unrelated**  **Definitely not related** | There is no evidence of any causal relationship to the medical device | Where relationship to the device and/or procedure can be excluded. | Temporal relationship of the onset of the event, relative to administration of the product, is not reasonable or another cause can by itself explain the occurrence of the event | There is no evidence of any causal relationship. | The AE is clearly not related to the study procedures (i.e., another cause of the event is most plausible and/or a clinically plausible temporal sequence is inconsistent with the onset of the event). | No relationship with the therapy, research assessment or therapy system. Other factor(s) certainly causative. |
| **Unlikely**  **Probably unrelated**  **Probably not related** | The relationship with the use of the investigational medical device seems not relevant and/or the event can be reasonably explained by another cause | Where the relationship with the use of the device seems not relevant and/or the AE can be reasonably explained by another cause, but additional information may be obtained. | Temporal relationship of the onset of the event, relative to administration of the product, is likely to have another cause which can by itself explain the occurrence of the event. | There is little evidence to suggest there is a causal relationship (e.g. the event did not occur within a reasonable time). There is another reasonable explanation for the event (e.g. the patient’s clinical condition, other concomitant treatment). | -- | -- |
| **Possibly**  **Possible**  **Possibly related** | The relationship with the use of the device is weak but cannot be ruled out completely | Where the nature of the event, underlying medical condition, concomitant medication or temporal relationship make it possible that the AE has a causal relationship to the device. Cases where relatedness cannot be assessed or no information has been obtained, shall also be classified as possible. | Temporal relationship of the onset of the event, relative to administration of the product, is reasonable but the event could have been due to another, equally likely cause. | There is some evidence to suggest a causal relationship. However, the influence of other factors may have contributed to the event (e.g. the patient’s clinical condition, other concomitant treatments). | An event that follows a reasonable temporal sequence from the initiation of study procedures, but that could readily have been produced by a number of other factors. | Possible relationship with therapy, research assessment or therapy system. Other factor(s) possibly causative. |
| **Probable**  **Probably related** | The relationship with the investigational medical device seems relevant and/or the event cannot be reasonably be explained by another cause | Where relationship with use of the device seems relevant and/or the AE cannot reasonably be explained by another cause, but additional information may be obtained. | Temporal relationship of the onset of the event, relative to the administration of the product, is reasonable and the event is more likely explained by the product than any other cause. | There is evidence to suggest a causal relationship and the influence of other factors is unlikely. | -- | -- |
| **Related**  **Definitely related**  **Causal relationship** | The serious event is associated with the investigational medical device beyond reasonable doubt | Where the AE is associated with the device and/or procedure beyond reasonable doubt | temporal relationship of the onset, relative to administration of the product, is reasonable and there is no other cause to explain the event, or a re-challenge (if feasible) is positive | There is clear evidence to suggest a causal relationship and other possible contributing factors can be ruled out. | The AE is clearly related to the study procedures. | Temporal relationship of the onset of the event, relative to occurrence of therapy, research assessment or therapy system, is reasonable and there is no other cause to explain the event. |

**Supplementary material S5. Examples of Adverse Events**

Examples of Adverse Events

- - Participant has a non-life threatening accidental injury (e.g. participant breaks leg, participant startled/surprised and sustains minor injury from reaction (back sprain)).
  - Non-life threatening self-harm unrelated to trial (e.g., goes to reservoir with intention to walk in; goes to multi-storey car park with intention to jump – but is diverted/changes mind).
  - Participant presents to AE, examined/ treated, but is not admitted.

Examples of Adverse Reactions

- - Participant states that the questions asked during an assessment were upsetting, which led the assessment being stopped early.
  - Participant has a panic attack as a result of talking about past experiences when they were asked what they felt caused them to experience psychosis.
  - Participant becomes paranoid as a result of inputting information about symptoms into the app.
  - App stops working and participant becomes distressed and requires additional support from a member of the care team.
  - Participant finds the app alerts intrusive, annoying but doesn’t experience a deterioration in mental health as a result (e.g. decides to mute alerts)

Examples of Serious Adverse Events

- - Participant is involved in a car accident/ other incident and requires surgery.
  - Participant experiences physical health change which requires an inpatient stay on a medical ward (e.g., pregnancy complication; Norovirus infection; injury following seizure brought on by drug misuse).
  - Participant has medication changed, which results in re-hospitalisation.
  - Participant takes an overdose and presents themselves to A&E, but this is not judged to be related to study procedures or interventions.

Examples of SARS/SUSARS

- - Participant experiences increased paranoia as a result of inputting information about symptoms into an app, which results in presentation to A&E/hospitalisation.
  - Participant becomes distressed as a result of participating in an assessment about their experiences and, as a direct result, self-harms.

**Supplementary material S6. Example report forms and logs**

*Adverse Event report form*

**[Study title] Adverse Events report form**

Please return completed reports to [name], [email address]

| **Details of Chief Investigator** | | |
| --- | --- | --- |
| **Name:** | |  |
| **Address:** | |  |
| **Email:** | |  |
| **Details of the Study** | | |
| **Full title of the study:** |  | |
| **Name of REC/IRB:** |  | |
| **REC/IRB reference number:** |  | |
| **IRAS ID:** |  | |
| **Research sponsor:** |  | |

| **Study Information** | | | |
| --- | --- | --- | --- |
| **Protocol Number:** |  | **Participant ID:** |  |
| **Site Name:** |  | **Event reported by/submitter:** |  |
|  |  |  |  |

| **Adverse Event** | | | | | | | | | | | | | | | | | | | | | | | | | |
| --- | --- | --- | --- | --- | --- | --- | --- | --- | --- | --- | --- | --- | --- | --- | --- | --- | --- | --- | --- | --- | --- | --- | --- | --- | --- |
| **NOTE: clinically significant abnormal lab results should be considered AEs** | | | | | | | | | | | | | | | | | | | | | | | | | |
| **Date of Report:** | | | | ____/____/______  DD MM YYYY | | | | | | | | | **Type of Report:** | | | | | | Initial  Follow Up **#**_____ | | | | | | |
| **Adverse Event Term:**  **[e.g. SAE, AE, AR]** | | | | ___________________________________________________________ | | | | | | | | | | | | | | | | | | | | | |
| **Date and time of Onset of Symptoms/Start of AE:** | | | | ____/____/______  DD MM YYYY | | | | | | | |  | | **_____:_____**  HH **:** MM | | | | | | | ** **Time unknown | | | | |
| **Resolution date and time:** | | | | ____/____/______  DD MM YYYY | | | | | | | |  | | **_____:_____**  HH **:** MM | | | | | | | ** **Time unknown | | | | |
| **Initial Date Reported to Sponsor:** | | | | ____/____/______  DD MM YYYY | | | | | | | |  | | **Date and time PI Aware of AE:** | | | | | | | ____/____/______  DD MM YYYY  **_____:_____**  HH **:** MM | | | | |
| **Participant Outcome:** | | | | | Resolved [recovered] | | | | | | | | |  | | Death | | | | | | | |  | |
|  | | | | | Resolved [recovered] with sequelae | | | | | | | | |  | | Unknown | | | | | | | |  | |
|  | | | | | Ongoing [not recovered/resolved] | | | | | | | | |  | |  | | | | | | | |  | |
| **Action Taken – Participant:** | | | | Continued Study | | | | | | | | | |  | | | Discontinued Study | | | | | | |  | |
| If discontinued, was it due to **this** AE? | | | | | | | | | | | | | | | | | Yes | | |  | | | No |  | |
| **Seriousness Classification** | | | | | | | | | | | | | | | | | | | | | | | | |  |
| **Serious Adverse Event:** | | | | | | **Yes**  **No** | | | | | If **Yes**, report to Sponsor within [x hours/days] and complete Seriousness criteria section below  If **No**, do not complete Seriousness Criteria section below | | | | | | | | | | | | | |  |
| **Seriousness Criteria:** (please tick at least one box and provide additional information if required for that criterion) | | | | | | | | | | | | | | | | | | | | | | | | |  |
|  | Death | Date: | ____/____/______  DD MM YYYY | | | | | | | Cause: | | | | | ___________________________________ | | | | | | | | | |  |
|  | Life threatening illness or injury | | | | | | | | | | | | | | | | | | | | | | | |  |
|  | Permanent impairment of a body structure or a body function (disability or incapacity) | | | | | | | | | | | | | | | | | | | | | | | |  |
|  | Medical or surgical intervention to prevent life-threatening illness or injury or permanent impairment to a body structure or a body function (disability or incapacity) | | | | | | | | | | | | | | | | | | | | | | | |  |
|  | In-patient / Prolonged Hospitalisation: | | | | | | Admit Date: | | ____/____/______  DD MM YYYY | | | | | | | | | Disch. Date: | | | | ____/____/______  DD MM YYYY | | |  |
|  | Related to: | | | | | |  | | Mental health | | | | | | | | |  | | | | Physical health | | |  |
|  | Led to foetal distress, foetal death or a congenital abnormality or birth defect | | | | | | | | | | | | | | | | | | | | | | | |  |
|  | Other (Please Specify): | | | | | | | _____________________________________________________ | | | | | | | | | | | | | | | | |  |

| **Causality, Severity, and Expectedness** | | | | | | | | | | | | | |
| --- | --- | --- | --- | --- | --- | --- | --- | --- | --- | --- | --- | --- | --- |
| **Relationship to Study Device/Digital Health Tool (software related)]:** | Not Related |  | Unlikely | |  | Possibly | |  | Probably |  | | Definitely |  |
| **Relationship to Study Hardware (handset, network}:** | Not Related |  | Unlikely | |  | Possibly | |  | Probably |  | | Definitely |  |
| **Relationship to Study Procedure:** | Not Related |  | Unlikely | |  | Possibly | |  | Probably |  | | Definitely |  |
| **Relationship to Therapy:** | Not Related |  | Unlikely | |  | Possibly | |  | Probably |  | | Definitely |  |
| **Relationship to Research Assessment:** | Not Related |  | Unlikely | |  | Possibly | |  | Probably |  | | Definitely |  |
| **Relationship to Remote Delivery (if applicable)** | Not Related |  | Unlikely | |  | Possibly | |  | Probably |  | | Definitely |  |
| **Severity Grade:** | | | | Mild | | |  | Moderate | | |  | Severe |  |
| **Expectedness:** | | | | Anticipated | | |  | Unanticipated | | |  |  |  |

| **Event Narrative [provide a description of the event]** | | | | | | | |
| --- | --- | --- | --- | --- | --- | --- | --- |
| (e.g. onset of symptoms, treatment, medications, outcome, reason for causality assessment) | | | | | | | |
| **_________________________________________________________________________________** | | | | | | | |
| _________________________________________________________________________________ | | | | | | | |
| _________________________________________________________________________________ | | | | | | | |
| _________________________________________________________________________________ | | | | | | | |
| _________________________________________________________________________________ | | | | | | | |
| **Relevant Medical History:** (please state any relevant pre-existing conditions) | | | | | | | |
| **_________________________________________________________________________________** | | | | | | | |
| _________________________________________________________________________________ | | | | | | | |
| _________________________________________________________________________________ | | | | | | | |
| _________________________________________________________________________________ | | | | | | | |
| **Where did the event take place?** | | | | | | | |
| In therapy |  | At home |  | [insert other study/trial information here] |  | Other |  |

| **Details of team discussion:** |
| --- |
| (To include date of discussion, who was present and decision) |
| **_________________________________________________________________________________** |
| _________________________________________________________________________________ |
| _________________________________________________________________________________ |
| _________________________________________________________________________________ |
| _________________________________________________________________________________ |

| **Guidance from Study Oversight Committee (e.g. Data Monitoring and Ethics Committee)** |
| --- |
| **_________________________________________________________________________________** |
| _________________________________________________________________________________ |
| _________________________________________________________________________________ |
| _________________________________________________________________________________ |
| _________________________________________________________________________________ |

| **Follow-up (if not resolved at time of completing from)** |
| --- |
| **_________________________________________________________________________________** |
| _________________________________________________________________________________ |
| _________________________________________________________________________________ |
| _________________________________________________________________________________ |
| _________________________________________________________________________________ |

| **Investigator Signature:** | | | | | | |
| --- | --- | --- | --- | --- | --- | --- |
|  |  |  |  |  |  |  |
|  | Name |  | Signature |  | Date (dd/mm/yyyy) |  |
|  |  | | | | | |
|  | Date reported to sponsor/REC: | | | | | |

*Device Deficiency form*

Please return completed reports to [name and contact details that form should be sent to for this study]

| **Study Information** | |
| --- | --- |
| **Protocol Number:** |  |
| **Investigational Device Name:** |  |
| **Investigator Name:** |  |
| **Site Name:** |  |

| **Participant & Device Information** | |
| --- | --- |
| **Participant ID:** |  |
| **Device Available for Evaluation?** |  |

| **Incident Information** | |
| --- | --- |
| **Performance endpoint:** |  |
| **Type of Incident:** | **Device Malfunction** |
|  | **Manufacture Defect** |
|  | **Labelling** (insufficient / inadequate instructions) |
|  | **Use Error** |
|  | **Procedure** (deployment, implantation, installation) |
| **Did the incident result in an Adverse Device Effect (ADE)?** (see section 2 for definition) | ** Yes No**  **If Yes, please record:**  **ADE term: ___________________________________**  **Onset date: ___________________** |
| **If there was an ADE, was the incident Serious?** | ** Yes –** complete the Safety Event Report Form and return to the PV office  ** No –** record the incident in the CRF / eCRF only |
| **Could this incident have led to an SAE if suitable action had not been taken, or intervention had not been made or circumstances had been less fortunate?** | ** Yes –** complete the “Incident that may have led to an SAE” Section  ** No** |
| **Detailed Description of Incident:**  **Please Complete For All Device Deficiencies**  (Attach separate sheets if necessary) |  |

| **Incident that May Have Led to an SAE** | |
| --- | --- |
| **Date and Time of Event Onset:** |  |
| **Action / Treatment / Outcome:** |  |
| **Related to Procedure:** | ** Related** |
|  | ** Not related** |
|  | ** Possibly related** |
| **Related to Investigational Device:** | ** Related** |
|  | ** Not related** |
|  | ** Possibly related** |
| **Event Status:** | ** Resolved** |
|  | ** Resolved with Sequelae** |
|  | ** Ongoing** |
|  | ** Death** |
|  | ** Unknown** |
| **Date and Time of Event Resolution:** |  |
| **Date reported to Sponsor/REC:** |  |

| **Investigator Signature:** | | | | | | |
| --- | --- | --- | --- | --- | --- | --- |
|  |  |  |  |  |  |  |
|  | Name |  | Signature |  | Date (dd/mm/yyyy) |  |

*Adverse Event log*

Please only enter one AE per row. Please ensure all sections are completed or item selected

| **Event No.** | **Briefly Describe the Adverse Event** | **Start Date**  **(DD/MM/YY)** | **End Date**  **(DD/MM/YY)** | **Seriousness** | | **Causality** | | | **Expectedness** | **Severity** | **Action Taken** | **Outcome** |
| --- | --- | --- | --- | --- | --- | --- | --- | --- | --- | --- | --- | --- |
|  |  |  |  | **Is this a SAE (Yes/No)** | **Seriousness criteria** | **Related to study hardware** | **Related to study software (e.g. CONNECT app)** | **Related to study procedure** |  |  |  |  |
|  |  |  |  | Yes*  No | Death  Life threatening illness or injury  Permanent impairment of a body structure of body function (disability or incapacity)  Medical or surgical intervention to prevent life-threatening illness or injury of permanent impairment to a body structure of body function (disability or incapacity)  In-participant/prolonged hospitalisation  Led to foetal distress, foetal death or a congenital abnormality or birth defect  Other (specify) | Not related  Unlikely related  Possibly related  Probably related  Definitely related | | | Anticipated  Unanticipated | Mild  Moderate  Severe | Continued study  Discontinued study  If discontinued, was it due to this AE? Yes/No | Resolved (recovered)  Resolved (recovered) with sequalae  Death  Unknown |
|  |  |  |  |  |  |  |  |  |  |  |  |  |
|  |  |  |  |  |  |  |  |  |  |  |  |  |
|  |  |  |  |  |  |  |  |  |  |  |  |  |

*If yes, report to sponsor and Research Ethics Committee

**Not related**– temporal relationship not reasonable or event explained in isolation by another cause 
**Unlikely related** – temporal relationship unlikely or event likely to be explained by another cause 
**Possibly related** – temporal relationship is reasonable but event could be due to another equally likely 
**Likely related**– temporal association is reasonable and event is more likely to be due to study intervention than other cause

**Definitely related**– temporal relationship is reasonable and there is no other cause to explain event, or re‐challenge is positive

**Supplementary material S7. Example reporting flowchart.**


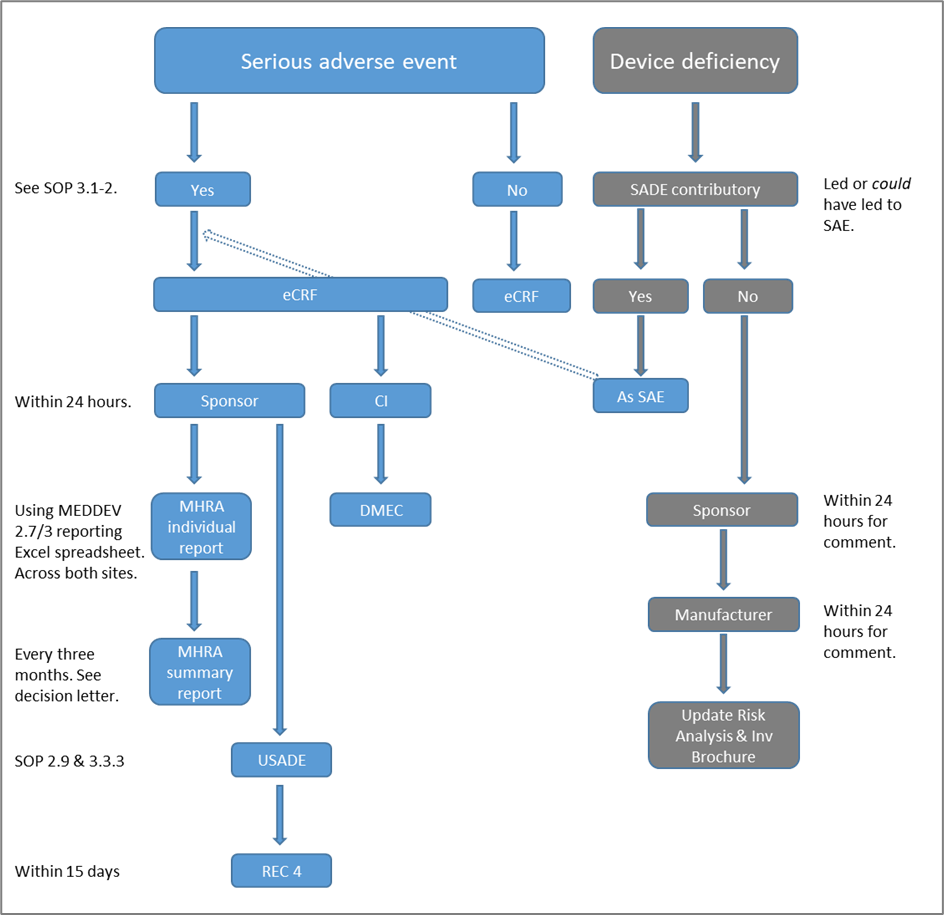


*SOP, Standard Operating Procedure; SADE, Serious Adverse Device Effect; SAE, Serious Adverse Event; eCRF, electronic Case Report Form; CI, Chief Investigator; MHRA, Medicines and Healthcare products Regulatory Agency; DMEC, Data Monitoring and Ethics Committee; USADE, unanticipated serious adverse device effect; REC, Research Ethics Committee.*

**Supplementary material S8. Example declaration form for study staff**

I confirm that I have read the document entitled “Standard Operating Procedures for Adverse Events in the [study name] study”, produced and maintained by the research group.

| **Study Personnel (PRINT)** | **Signature** | **Initials** | **SOP version** | **Date** |
| --- | --- | --- | --- | --- |
|  |  |  |  |  |
|  |  |  |  |  |
|  |  |  |  |  |
|  |  |  |  |  |
|  |  |  |  |  |
|  |  |  |  |  |
|  |  |  |  |  |
|  |  |  |  |  |
|  |  |  |  |  |
|  |  |  |  |  |
|  |  |  |  |  |
|  |  |  |  |  |
|  |  |  |  |  |
|  |  |  |  |  |
|  |  |  |  |  |
|  |  |  |  |  |

**I confirm that the above noted individual(s) are appropriately qualified to perform the delegated standard operating procedure.**

Head of Research Unit signature:

**Supplementary material S9. Example glossary of terms section**

| Term | definition |
| --- | --- |
| adverse device effect (ADE) ^[[2]](#footnote-2)^ | Adverse event that is deemed to be related to the use of an Investigational Medical Device. This definition includes adverse events resulting from insufficient or inadequate instructions for use, deployment, [implantation], installation, or operation, or any malfunction of the Investigational Medical Device. This definition includes any event resulting from use error or from intentional misuse of the Investigational Medical Device. Use error refers to an act or omission of an act that results in a different device response than intended by the manufacturer or expected by the user |
| adverse event (AE)2 | An unexpected medical problem that happens during treatment with a drug or other therapy. Adverse events may be mild, moderate, or severe, and may be caused by something other than the drug or therapy being given. |
| adverse reaction (AR) 2 | Any adverse event judged by either the reporting investigator or the sponsor as having a reasonable causal relationship (e.g. definitely, probably or possibly related) to an investigational psychological therapy or other intervention, [investigational medial product, or study procedure] related to any dose or duration of therapy administered to that subject. |
| anticipated serious adverse device effect (ASADE) 2 | A serious adverse device effect which by its nature, incidence, severity or outcome has been previously identified in the risk analysis report, clinical hazard log, Clinical Investigational Plan, protocol, or the Investigator’s Brochure. [state whether or not any ASADEs are expected in the current study/trial]. |
| causality | Determining the relationship between the suspect product (i.e., drug, device, therapy, intervention), and an adverse event. |
| chief investigator (CI) | The lead researcher for a research project (responsible for the conduct of the whole project). |
| CLINICAL TRIAL | A research project that compares two or more treatments in patients with a particular condition or at risk of a condition to help generate high quality evidence about which is the more effective treatment or preventative strategy. |
| Data Monitoring and Ethics Committee (DMEC) | To safeguard the interests of trial participants, monitor the main outcome measures including safety and efficacy, and monitor the overall conduct of the study/trial. |
| DIRECTORATE GENERAL OF HEALTH SERVICES (india) | A repository of technical knowledge concerning Public Health, Medical Education and Health Care. |
| DRUGS CONTROLLER GENERAL (INDIA) | The head of department of the Central Drugs Standard Control Organization of the Government of India responsible for approval of licences of specified categories of drugs such as blood and blood products, IV fluids, vaccines, and sera in India. |
| Expert by experience | People with experience of using services as either a service user or a carer who are interested in undertaking research activities. |
| investigator’s brochure | A compilation of the clinical and nonclinical data on the investigational product(s) that are relevant to the study of the product(s) in human subjects. Its purpose is to provide the investigators and others involved in the trial with the information to facilitate their understanding of the rationale for, and their compliance with, many key features of the protocol, such as the dose, dose frequency/interval, methods of administration: and safety monitoring procedures. |
| Medical device/ investigational medical device | A medical device can be any instrument, apparatus, implement, machine, appliance, implant, reagent for in vitro use, software, material or other similar or related article, intended by the manufacturer to be used, alone or in combination for a medical purpose.    A medical device being assessed for safety or performance in a clinical investigation. This includes medical devices already on the market that are being evaluated for new intended uses, new populations, new materials or design changes. |
| Peer researcher | People with lived experience of the issues being studied take part in directing and conducting the research. |
| principal researcher (PI) | The person(s) in charge of a clinical trial or a scientific research grant. The principal investigator prepares and carries out the clinical trial protocol (plan for the study) or research paid for by the grant. The principal investigator also analyses the data and reports the results of the trial or grant research. |
| serious adverse event (SAE) 2 | An adverse event is defined by as serious if it:   1. Results in death or, 2. Is a life-threatening illness or injury or, 3. Requires [voluntary/involuntary] hospitalisation or prolongation of existing hospitalisation or, 4. Results in persistent or significant disability or incapacity or, 5. Medical or surgical intervention required to prevent any of the above, 6. Leads to foetal distress, foetal death or consists of a congenital anomaly or birth defect or, 7. Is otherwise considered medically significant by the investigator. |
| sponsor | A person, company, institution, group, or organization that oversees or pays for a clinical trial and collects and analyses the data. |
| suspected unexpected serious adverse reaction (SUSAR) 2 | An adverse event that is judged to be all of the following:   1. Serious (see SAE definition) 2. Possibly, probably or definitely related (see AR definition) 3. Unexpected (see UAR definition) |
| unanticipated serious adverse device effect (USADE) 2 | Serious Adverse Device Effect which by its nature, incidence, severity or outcome has not been identified in the current version of the risk analysis report, clinical hazard log, Clinical Investigational Plan, protocol, or the Investigator’s Brochure. |

1. These are the most prominent international guidelines on AE monitoring, recording and safety reporting for pharmacological trials (ICH) and medical devices (ISO). The ICH and ISO definitions of what constitutes an AE/SAE are summarised in the table. The ICH and ISO guidelines typically form the basis of national guidelines and regulations (e.g. MHRA, HRA, NHMRC, FDA, EU Clinical Trials Regulation 536/2014). Namely, in line with the ICH guidelines, AEs must be classified to determine whether they are **serious**, **related** to the intervention, and/or **unexpected** (see Figure 1), with AEs that meet all three criteria (serious, related and unexpected) then requiring further safety reporting. [↑](#footnote-ref-1)
2. The researchers should update the table with the specific Adverse Event definitions used in their study [↑](#footnote-ref-2)
