# Supplementary material for: Measurement of Adverse Events in Studies of Digital Health Interventions for Psychosis: Guidance and Recommendations Based on a Literature Search and Framework Analysis of Standard Operating Procedures
Source: Schizophr Bull. 2024 Apr 29;50(6):1456–70. doi: 10.1093/schbul/sbae048 (PMC11548926; doi:10.1093/schbul/sbae048)
Supplement: sbae048_suppl_Supplementary_Methods [file sbae048_suppl_supplementary_methods.docx]

**Supplementary Methods**

***Supplementary methods SM1: Additional details of literature search***

*Overview of search strategy*

For pragmatic reasons, we combined searches from two previous projects led by members of our network. All searches were restricted to peer-reviewed articles published in English between January 2010 and the present day with human participants. The symbols ‘?’ and ‘*’ are wildcards representing one (‘?’) or more (‘*’) characters, respectively.

*Search 1: Barriers and Facilitators review search (search date: 11/10/21)*

Seven databases were searched (MEDLINE, PsycINFO, PsycARTICLES, Embase, Health and Psychosocial Instruments, PubMed, Web of Science). As this search was from a review relating to barriers and facilitators of engagement with digital health tools across SMI diagnoses, it included additional search terms (e.g. bipolar). Overall search terms were as follows:

(schizophren* or schizo?affective or psychotic or psychos?s or paranoia or paranoid or hallucination* or delusion* or bipolar or SMI or serious mental illness or severe mental illness or manic or mania or grandios* or serious mental health or severe mental health or EIS or Early intervention service? or Early intervention team? or early intervention program? or CMHT? or community mental health team? or assertive outreach team? or AOT? or home treatment team? or home based treatment team? or crisis team?).ti,kw,ab.

AND

(digital or m?health or e?health or phone? or smart?phone? or mobile? or app? or application? or wearable? or online or internet or cyber or web or mtherap* or etherap* or Technology or technologies or technological or webpage or website or smart?watch or smart?watches or AI or artificial intelligence or machine learning or smart?device? or digital phenotyping or digital phenotype? or sensing or passive data or passive monitoring or GPS or global positioning system or gyroscope? or accelerometer?).ti,kw,ab.

AND

(barrier? or facilitator? or enabler? or obstacle or implement* or engage* or adhere* or satisfaction or acceptability or usability or user experience? or satisfaction or usage or adoption or user perspective? or qualitative or interview or focus group or feasibility or Co-design).ti,kw,ab.

*Search 2: Virtual Reality study search (search date: 29/04/22)*

Seven databases were searched (MEDLINE, PsycINFO, PsycARTICLES, Embase, Health and Psychosocial Instruments, PubMed, Web of Science) using the following terms:

(schizophren* or schizo?affective or psychotic or psychos?s or paranoia or paranoid or hallucination* or delusion*).ti,kw,ab.

AND

(virtual reality or VR).ti,kw,ab.

***Supplementary methods SM2: PICO criteria***

*Participants/population*

Included

- People aged 16 or over with psychosis or a schizophrenia spectrum diagnosis (e.g. schizophrenia, schizoaffective, schizophreniform, psychosis not otherwise specified).

*Intervention*

Included

- Studies testing the actual use of digital health tools that aim to monitor or improve the mental or physical health of people with a psychosis or schizophrenia spectrum diagnosis
- Delivered using a device such as a smartphone app, text messaging, online/website, virtual reality (VR), or wearable device.

Excluded

- Digital tools used as a component during in-person sessions with no remote use outside these sessions*
- Digital tools used purely for research purposes (with no likely eventual clinical application)
- Only included video-conferencing or phone calls
- Served only as an appointment booking system for in-person therapy
- Electronic health records that only health professionals could view and contribute to
- Only used to screen for the presence of a mental health condition
- Harvested existing data from electronic health records or mainstream social media to make predictions or classifications with regards to mental health.

*With the exception of VR studies, which were included even if they were used during in-person sessions only. The rationale for this was that VR headsets are not commonly owned by the general public but may be in the future. Studies to date are therefore unlikely to include remote use but this is something that may be done in the future.

Comparison

- No comparison group needed

Outcomes

- No specific outcome needed

***Supplementary methods SM3: instructions for supplying SOP***

Authors who agreed to provide the SOPs/procedure/guide they used to collect AE data in a relevant study were directed to upload the relevant information via a web-based proforma using the Qualtrics survey system, according to the following instructions:

*“For some studies, the Standard Operating Procedure (SOP) might be a specific document that provides additional, detailed guidance on how Adverse Events should be monitored and reported in the study; for other studies this information might be covered in a specific sub-section of the protocol or a sub-section of another document. We are interested in any study document that gives guidance on how Adverse Events are monitored/reported. If this is a sub-section of a larger document, you can just upload the relevant part”.*

***Supplementary methods SM4: details of data analysis and synthesis***

*Overview*

As described in the main manuscript, we used the existing headings and sub-headings of the EMPOWER SOP both as the initial headings and sub-headings of the template SOP and to create our *a priori* coding framework. This allowed us to draft an overall collated template SOP and simultaneously deductively code the contents of other SOPs into the framework. Where headings or sub-headings from other SOPs were not present in the EMPOWER SOP, we also added extra headings or sub-headings in the template SOP and coded inductively as appropriate. Further details of these processes are described below, with numbers indicating tasks that were conducted simultaneously between the template SOP drafting and framework analysis.

*Template SOP*

1. **Familiarisation phase.** Authors EE and CR first read all collated documents (from DHI studies, funders/regulators, and UK NHS Trusts or universities) to familiarise themselves with the data and to identify all sections of the documents related to how AEs are elicited, measured, monitored, recorded, or reported. Typically, for SOPs provided by DHI studies and from UK NHS Trusts and universities, the whole document was relevant. Funder/regulator documents often covered a range of other aspects of research governance or methods, with only a sub-set of sections relevant to AEs monitoring. In this case, only the AE-related sections were subsequently considered for inclusion in the template SOP or coded in the framework analysis.
2. **Initial template SOP.** An initial template SOP was created, which consisted of the EMPOWER SOP’s headings, sub-headings and content, with details that were specific to the EMPOWER study only (e.g., the study name, sponsor, funder, chief investigator) removed and replaced with generic field codes as appropriate (i.e., “<study name>”, “<study sponsor>”, “<study funder>”, “<chief investigator>”).
3. **Populating the template SOP (deductive).** Relevant text was copied and pasted from each new SOP document into the relevant section and sub-section of the template SOP.
4. **Populating the template SOP (inductive).** New sections or sub-sections were added to the template SOP as needed and relevant information copied into these sections.
5. **Summary and synthesis.** Once all relevant information had been copied into the draft template SOP, elements that were similar across SOPs were combined to avoid repetition. However, in some cases it was informative to retain multiple alternative examples (e.g., examples of expected AEs) from across SOPs to illustrate possible considerations across different DHI types or study designs. A preface was added to the template SOP instructing researchers using the template to retain examples/sections of relevance to their particular study/DHI design and delete irrelevant examples/sections. Similarly, given that the included SOPs represented a mixture of medical device studies (e.g., EMPOWER) and studies testing a DHI that is not registered as a medical device, some content differed substantially between SOPs. In these cases, alternative sections were presented for medical device studies and non-medical device studies, respectively. The main example of this was the SOP section listing definitions of relevant AE sub-types – this section was presented separately for medical device studies (relevant definitions: Adverse Device Effect (ADE), Serious Adverse Device Effect (SADE), etc) and studies testing a DHI that was not registered as a medical device (relevant definitions: Adverse Reaction (AR), Serious Adverse Reaction (SAR), etc). Again, the preface instructed researchers using the template to select only the section relevant to their particular study.
6. **Iteration with additional data.** The process was repeated to add in extra information about AE procedures (how AEs were elicited; how staff were trained/supervised) and definitions gathered during Phase 3.

*Framework analysis*

1. **Familiarisation phase.** As above.
2. **Initial coding framework.** The headings and sub-headings of the EMPOWER SOP were used as the codes and sub-codes of the coding framework. A coding matrix (table) was created in a Microsoft Excel document, with each of these initial codes and sub-codes represented by a column and each SOP document represented by a row.
3. **Deductive coding.** Authors EE and CR read all sections of the SOP documents that had been identified as relevant to AEs procedures during the familiarisation phase and coded these, section-by-section, into the framework matrix. Practically speaking, this involved summarising text from the specific SOP in the relevant cell of the framework matrix (i.e., the cell corresponding to the relevant code and that specific SOP document). Where definitions of AE subtypes were coded (e.g., AE, SAE, SAR, SADE), the entire definition was copied into the relevant cell, to allow detailed comparison and analysis of specific wording on the definitions across SOPs.
4. **Inductive coding.** Where sections of the SOP being analysed did not fit within the initial codes and sub-codes, a new column was created in the coding matrix to accommodate this new code/sub-code. Relevant content from all SOPs was copied into the relevant cell(s) of the new column.
5. **Summary and synthesis**. Once the whole dataset had been coded, each deductive and inductive code and sub-code was systematically examined by re-reading all data coded against it (i.e., data in that column of the framework matrix) from across all SOPs. Key similarities and differences across SOPs were examined and summarised descriptively. Where similar information was present across SOPs, this was synthesised to give an overall description of the central ideas present.
6. **Iteration with additional data.** As above.

***Supplementary methods SM5: email template requesting additional information***

Dear [name],

In Writing Group 2 of the iCharts network, we have been working hard to analyse and collate standard operating procedures (SOPS) for Adverse Events (AEs) reporting. We have gathered SOPS from across a number of studies as well as from other sources such as funders and regulators. We are in the process of harmonising these into a central template document that can be used to help guide future AEs reporting.

We have noticed that some aspects of AEs reporting procedures are not documented in the SOPS. (e.g. the procedure for training research staff to ask about/document/report AEs). Whilst we are aware anecdotally that research staff are being trained in how to ask about and report AES, these procedures and methods are not always explicitly documented in the SOP.

In the interest of capturing and developing best practice guidelines for training research staff in AE reporting, we wondered if you would be open to sharing your procedure or approach to training research staff and capturing AEs.

For example, the overarching question we have in mind is: ***How do research assistants or trial therapists (or others) elicit/monitor AEs on a day-to-day basis in your study?*** Specific questions we have in mind are:

- How are research assistants or trial therapists (or others) trained and supervised in eliciting/monitoring Adverse Events? Is there a specific training session on this topic when you induct/train staff on your study? If so, would you be willing to share the slides or training material with us to integrate in our reporting guidelines?
- Do research staff receive specific ongoing supervision on asking about /documenting / reporting AEs?
- Are AEs kept on their radar in other ways (e.g. as a standing agenda item in weekly/monthly team/project meetings)?
- Does the PI / supervisor regularly ask about AEs in supervision meetings with research staff?
- Do research staff record AEs that are spontaneously reported by participants, or do they ask open question(s) about whether the participant has experienced any AEs? (or both). If so, what question(s) are asked and how often / when are they asked?
- Do research staff use a structured questionnaire measure to monitor Adverse Events? If so, what questionnaire and how often is it administered?
- Do they screen casenotes for Adverse Events reports?
- Do they use another method not listed here?

If you could provide a few sentences describing how AEs are initially captured in your study that would be very much appreciated and helpful.

Thank you in advance.

Best wishes,

Emily and Cara

*SIRS Research Harmonisation Group 2021-2023, Writing Group 2 co-leads*
